# *Lactobacillus*-derived extracellular vesicles counteract Aβ42-induced abnormal transcriptional changes through the upregulation of MeCP2 and Sirt1 and improve Aβ pathology in Tg-APP/PS1 mice

**DOI:** 10.1038/s12276-023-01084-z

**Published:** 2023-09-13

**Authors:** Hyejin Kwon, Eun-Hwa Lee, So-Young Park, Jin-Young Park, Jin-Hwan Hong, Eun-Kyung Kim, Tae-Seop Shin, Yoon-Keun Kim, Pyung-Lim Han

**Affiliations:** 1 Department of Brain and Cognitive Sciences, Scranton College, Ewha Womans University, Seoul, 03760 Republic of Korea; 2 MD Healthcare Inc., Rm 1403 Woori Technology Bldg, World Cup Buk-Ro 56-Gil, Mapo-Gu, Seoul, 03923 Republic of Korea

**Keywords:** Alzheimer's disease, Biological therapy

## Abstract

Mounting evidence suggests that probiotics are beneficial for treating Alzheimer’s disease (AD). However, the mechanisms by which specific probiotics modify AD pathophysiology are not clearly understood. In this study, we investigated whether *Lactobacillus paracasei*-derived extracellular vesicles (*Lpc*-EV) can directly act on neuronal cells to modify amyloid-beta (Aβ)-induced transcriptional changes and Aβ pathology in the brains of Tg-APP/PS1 mice. *Lpc*-EV treatment in HT22 neuronal cells counteracts Aβ-induced downregulation of *Brain-derived neurotrophic factor (Bdnf)*, *Neurotrophin 3 (Nt3)*, *Nt4/5*, and *TrkB* receptor, and reverses Aβ-induced altered expression of diverse nuclear factors, including the downregulation of *Methyl-CpG binding protein 2 (Mecp2)* and *Sirtuin 1 (Sirt1)*. Systematic siRNA-mediated knockdown experiments indicate that the upregulation of *Bdnf, Nt3, Nt4/5*, and *TrkB* by *Lpc*-EV is mediated via multiple epigenetic factors whose activation converges on *Mecp2* and *Sirt1*. In addition, *Lpc*-EV reverses Aβ-induced downregulation of the Aβ-degrading proteases *Matrix metalloproteinase 2 (Mmp-2)*, *Mmp-9*, and *Neprilysin (Nep)*, whose upregulation is also controlled by MeCP2 and Sirt1. *Lpc*-EV treatment restores the downregulated expression of *Bdnf, Nt4/5, TrkB*, *Mmp-2, Mmp-9*, and *Nep*; induces the upregulation of MeCP2 and Sirt1 in the hippocampus; alleviates Aβ accumulation and neuroinflammatory responses in the brain; and mitigates cognitive decline in Tg-APP/PS1 mice. These results suggest that *Lpc*-EV cargo contains a neuroactive component that upregulates the expression of neurotrophic factors and Aβ-degrading proteases (*Mmp-2, Mmp-9*, and *Nep*) through the upregulation of MeCP2 and Sirt1, and ameliorates Aβ pathology and cognitive deficits in Tg-APP/PS1 mice.

## Alzheimer’s disease: bacteria-derived vesicles help to combat neurodegeneration

Small molecular membrane-bound vesicles released by probiotic bacteria can help to counteract the negative effects of amyloid-beta (Aβ), a protein that clumps inside the brain and is associated with the progression of Alzheimer’s disease. A team led by Yoon-Keun Kim from MD Healthcare Inc. and Pyung-Lim Han from Ewha Womans University, both in Seoul, South Korea, showed that these molecular packets — known as extracellular vesicles and derived from Lactobacillus bacteria found in the mouth and gut — can mitigate against Aβ-induced changes in gene expression in mouse neurons. By delivering neuroactive components, the packets also reversed cognitive decline, prevented further accumulation of Aβ and lessened inflammation in the brains of mice engineered to develop an Alzheimer’s-like disease. The findings help to explain the beneficial effects of probiotics in people with this devastating neurodegenerative condition.

## Introduction

Alzheimer’s disease (AD) is a neurodegenerative disease that causes Aβ-induced neuropathology and cognitive deficits^1^. While there are genetic cases of AD, the vast majority of cases occur sporadically in aged individuals^2^. As the global elderly population grows, a proper strategy for AD treatment is urgently needed. A key mechanism in AD pathology is the accumulation of Aβ in the brain. Aβ accumulation is accelerated by an imbalance of Aβ production and clearance^3,4^. Aβ accumulation produces various neuropathological changes, including increased oxidative stress, neuroinflammatory responses, decreased neurogenesis, synaptic and neuritic deterioration, and neuronal loss^1,5,6^. Transgenic mice overexpressing mutant forms of the human β-amyloid precursor protein (APP) and presenilin (PS) genes exhibit Aβ accumulation, neuroinflammatory responses in the brain, and cognitive decline^6,7^, supporting the notion that Aβ-induced change is a critical mechanism in AD pathology.

Emerging evidence supports the role of the gut microbiota in regulating brain function and the pathogenesis of AD. Multiple pathways have been proposed to explain the effects of gut microbiota, including activation of resident immune cells in the gut epithelium to release cytokines, production of bacterial metabolites and signaling molecules, changes in intestinal membrane permeability, and propagation of bacterial amyloid-seeding effects^8,9^. Recent studies, including our own, have demonstrated that extracellular vesicles (EVs) derived from *Lactobacillus plantarum* have neuroactive potential to reverse stress-induced downregulation of MeCP2, Sirt1, and neurotrophic factors in hippocampal neurons and to improve stress-induced depressive behavior in mice^10,11^. *Lactobacillus paracasei*-derived EVs exert anti-inflammatory effects against intestinal inflammatory responses in dextran sulfate sodium (DSS)-induced colitis^12^. However, it has not been tested whether *Lactobacillus*-derived EVs have bioactive potential to modify Aβ-induced pathology and improve behavioral deficits.

Histone modifications, such as acetylation and methylation at the N-terminal region of histones, can change chromatin structures, the results of which promote or suppress the expression of specific genes^13,14^. Methyl-CpG binding protein (MeCP2) binds to methylated CpG dinucleotides, which can regulate gene expression negatively or positively, in concert with histone modification factors or other nuclear factors^15,16^. Various epigenetic mechanisms including MeCP2 play roles in the expression of neurotrophic factors^17–19^. MeCP2 deletion or MeCP2 knockdown downregulates *Bdnf* expression in the hippocampus of mice^20–22^. Conversely, MeCP2 overexpression in cortical neurons or in the hippocampus of mice upregulates *Bdnf* expression^23,24^. Tg-APP/PS1 mice have reduced levels of neurotrophic factors in the hippocampus, which is associated with MeCP2 downregulation^22^. Sirt1 knockdown downregulates the expression of *Bdnf, Nt3*, and *Nt4/5* in HT22 cells^10^. HDAC inhibitors and siRNA-mediated SUV39H1 knockdown upregulates BDNF expression^25,26^. BDNF levels are decreased in the hippocampus and cerebrospinal fluid (CSF) of patients with AD^27,28^. BDNF, NT3, and NT4/5 act through TrkB and TrkC^29^. Transgenic mice overexpressing Aβ have reduced expression levels of BDNF, whereas activation of TrkB receptors with BDNF mimetics improves cognitive decline in AD model mice^30,31^. These results suggest that proper targeting of epigenetic mechanisms could be a strategy to upregulate the expression of neurotrophic factors in the brain and to mitigate AD pathology in the brain.

In the present study, we investigated whether *Lactobacillus paracasei*-derived EVs can modify the expression of neurotrophic factors, and alleviate Aβ-induced pathology and cognitive decline in Tg-APP/PS1 mice.

## Materials and methods

### Animals

Tg-APPswe/PS1dE9 (Tg-APP/PS1) mice, which overexpress mutant forms of human amyloid precursor protein (APP) and presenilin-1 (PS1) genes, were maintained as C57BL6 × C3H F1 hybrids as previously described^22,32^. After weaning, male and female mice were separately housed with 2-3 animals in a standard clear plastic cage in a temperature (23–24 °C)- and humidity (50–60%)-controlled environment under a 12 h light/dark cycle (lights on from 07:00 to 19:00 h), with ad libitum access to water and food.

Genotyping of Tg-APP/PS1 mice was determined by genomic PCR using the following primers; 5′-CTAGGCCACAGAATTGAAAGATCT-3′ and 5′-GTAGGTGGAAATTCTAGCATCATCC-3′ for WT (324 bp), 5′-AATAGAGAACGGCAGGAGCA-3′ and 5′-GCCATGAGGGCACTAATCAT-3′ for the PS1 gene (324/608 bp), and 5′-AGGACTGACCACTCGACCAG-3′ and 5′-CGGGGGTCTAGTTCTGCAT-3′ for the APP gene (324/350 bp).

Tg-APP/PS1 mice were maintained and handled in accordance with The Animal Care Guidelines of Ewha Womans University, and the experimental procedure for EV treatment in this study was approved by the Ewha Womans University Animal Care and Use Committee (IACUC 16-019).

### Preparation of *Lactobacillus paracasei-*derived EVs

Bacterial culture and EV isolation were carried out as previously described^12^. In brief, *Lactobacillus paracasei* was cultured in MRS broth (MBCell, Seoul, Republic of Korea) for 18 h at 37 °C with gentle shaking (150 rpm). When the optical density of the culture reached 1.0 at 600 nm, the bacterial culture was centrifuged at 10,000 × *g* for 20 min, and the supernatant was collected and passed through a 0.22-μm bottle-top filter (Corning, NY, USA) to remove remaining cells or cell debris. The filtrate was concentrated using a MasterFlex pump system (Cole-Parmer, IL, USA) and a 100-kDa Pellicon 2 Cassette filter membrane (Merck Millipore, MA, USA) and was subsequently passed through a 0.22-μm bottle-top filter. *Lactobacillus paracasei-*derived EVs were obtained from the resulting filtrate by centrifugation at 150,000 × *g* for 3 h at 4 °C. Pellets were washed and resuspended in PBS (137 mM NaCl, 2.7 mM KCl, 10 mM Na_2_HPO_4_, and 1.8 mM KH_2_PO_4_). The protein concentration of the resuspended EV fractions was determined using a BCA protein assay kit (Thermo Fisher Scientific, MA, USA). Collected EVs were stored at −80 °C until use. EVs from *Lactobacillus paracasei* were named *Lpc*-EV.

### Aβ42 treatment in HT22 cells

HT22 cells, a hippocampus-derived neuronal cell line, were cultured as previously described^22,33^. HT22 cells were plated at a density of 1.0 × 10^5^ cells/well in a 6-well plate (SPL Life Science, Pocheon-si, Republic of Korea) and grown in DMEM (LM-001–05; Welgene, Gyeongsan, Republic of Korea) supplemented with 10% heat-inactivated fetal bovine serum (FBS; FB02–500, Serum Source International, Charlotte, NC, USA) and 1% penicillin (20 units/ml)/streptomycin (20 mg/ml) (LS020-02, Welgene).

Aβ42 (03112, Invitrogen, Camarillo, CA, USA; Sigma Aldrich) was dissolved in 5% DMSO to 400 μM, which was aliquoted into 10 μl samples, and stored at −80 °C until use. Each 10 μl of 400 μM Aβ42 aliquot was diluted to 10 μM in 0.4 ml of 1X PBS (137 mM NaCl, 2.7 mM KCl, 10 mM Na_2_HPO_4_, and 1.8 mM KH_2_PO_4_), and incubated at 4 °C for 24 h with gentle rocking. HT22 cells cultured in a 6-well plate were washed with DMEM containing 1% FBS, and then 1.8 ml of fresh DMEM containing 1% FBS without penicillin/streptomycin and 0.2 ml of 10 μM aged Aβ42 were added. Cells were incubated for 24 h and harvested for analyses.

### Transfection of siRNA-target genes and plasmid DNA into HT22 cells

Transfection of siRNA-target gene and plasmid DNA into HT22 cells was carried out as previously described^22,33^. Briefly, HT22 cells were plated at a density of 1.0 × 10^5^ cells/well in a 6-well plate containing 10% FBS. After 24 h, HT22 cells were washed with DMEM containing 1% FBS, and siRNA transfection was carried out using Lipofectamine-2000 (13778-075; Invitrogen, Carlsbad, CA, USA). Lipofectamine-2000 (9 μl) and 20 μM siRNA (3 μl) were separately diluted in 150 μl of Opti-MEM® Medium (31985070, Gibco, Thermo Fisher Scientific, Paisley, Scotland, UK). Diluted siRNA was mixed with Lipofectamine-2000 at a 1:1 ratio, and the mixture was incubated for 5 min at room temperature. The siRNA-lipid complex (250 μl) was gently pipetted onto HT22 cells in a 6-well plate, and the cells were incubated for 24 h. The final treatment dose was 20 μg of siRNA/7.5 μl of Lipofectamine-2000/well.

Control siRNA (siCON, SN-1012), MeCP2-siRNA (#1385135; NM_001081979.2), Sirt1-siRNA (#93759; NM_001159589.2), Sirt5-siRNA (#68346; NM_178848.3), Kdm4a-siRNA (#230674; NM_001161823.1), Hdac2-siRNA (#15182; NM_008229.2), Setdb1-siRNA (#84505; NM_001163641.1), and G9a-siRNA (#110147; NM_001286573.1) were purchased from Bioneer Co. (Daejeon, Republic of Korea). The siRNAs were resolved to 50 ng/μl in siRNA dilution buffer (B-002000-UB-100, Dharmacon, Lafayette, CO, USA).

To induce MeCP2 overexpression in HT22 cells, the pCNS-D2-MeCP2 plasmid DNA (0.125 μg) and Lipofectamine-2000 (10 μl) were separately diluted in 250 μl of Opti-MEM® Medium (31985070; Gibco-Thermo Fisher Scientific, Paisley, Scotland, UK) and incubated for 5 min at RT. Diluted plasmid DNA and Lipofectamine-2000 (250 μl each) were mixed at a 1:1 ratio and incubated for 20 min at room temperature. The plasmid DNA and Lipofectamine mix (500 μl) were dropped onto HT22 cells in each well of a 6-well plate containing 1.5 ml of DMEM (no FBS and no antibiotics). After 6 h, the medium was replaced with DMEM containing 1% FBS, and HT22 cells were grown further for 18 h. MeCP2 expression levels were determined using RT‒PCR at the indicated time points. Aβ42 (1 μM, final) or Aβ42 plus *Lpc*-EV (10 μg per ml, final) were treated starting 24 h after transfection. The pCNS-D2-MeCP2 plasmid was obtained from Korea Human Gene Bank, Medical Genomics Research Center, KRIBB, Republic of Korea.

### Microarray analysis

Microarray analysis was carried out as previously described^34–36^. Briefly, HT22 cells were plated at a density of 1.0 × 10^5^ cells/well in a 6-well plate. After 24 h, the cells were treated with Aβ42 (1 μM, final) or Aβ42 plus *Lpc*-EV (10 μg/ml, final) and incubated for an additional 24 h. Total RNA from HT22 cells was isolated using RNeasy Mini Kit columns (Qiagen, Hilden, Germany). The purified RNAs were quantified using a NanoDrop 1000 V3.7.1 Spectrophotometer (NanoDrop Technologies, Wilmington, DE, USA) and Agilent Bioanalyzer 2100 Expert (Agilent Technologies, Palo Alto, CA, USA).

Purified total RNA (400 ng) with a 28 S/18 S rRNA ratio of 1.8~2.0 and an A260/280 ratio of 1.8~1.9 was converted to first- and second-strand cRNA, which was then converted to biotin-labeled cRNA samples. Purified biotin-labeled cRNAs (7.5 µg) were fragmented in an array fragmentation buffer by heating to 94 °C for 35 min. Each of the fragmented, biotin-labeled cRNA samples (6.0 µg) was hybridized to a GeneChip™ Mouse Gene 2.0 ST Array, representing 33,793 mouse gene transcripts. After washing, the array signals were amplified with Amersham Fluorolink streptavidin*-*Cy3 (GE Healthcare Bio-Sciences, Little Chalfont, UK) and scanned using GeneChip® HT Scanner and AGCC software (Affymetrix GeneChip® Command Console, Version 3.2.2).

Microarray signals were converted into log2 scale values and normalized using a robust multi-array average (RMA) method implemented in Affymetrix® Power Tools (APT). The false discovery rate (FDR) was controlled by adjusting the p value using the Benjamini-Hochberg algorithm. The log_2_ values of microarray data were used to account for the differences in the expression levels. Macrogen Inc. (Seoul, South Korea) was requested to perform GeneChip hybridization and collect raw data, including signaling reading of the internal quality control probes and extraction of the scanned raw data.

### Gene Ontology enrichment analysis

Normalized microarray signal values between two experimental groups were ranked by signed log_10_-transformed t-test *p* values and analyzed using the Rank-Rank Hypergeometric Overlap (RRHO), as previously described^36,37^. A RRHO heatmap of microarray signal values was constructed using the RRHO website (http://systems.crump.ucla.edu/rankrank/). The ranked value was colored red if it was higher than the value of the comparison group, and blue if it was lower. A total of 33,793 signal values were analyzed and plotted on a RRHO geographic map. Genes that were up- or downregulated by Aβ and their expression was reversed by *Lpc*-EV were selected using a top 20% cutoff. Identified genes were grouped into functional clusters using *k*-means clustering. Gene Ontology (GO) enrichment analysis was used to determine whether any clusters assigned by *k*-means clustering contained genes that were involved in key biological processes, such as nervous system development or neurogenesis, regulation of DNA-binding transcription factors, chromatin modification and/or histone modification. The GO term hierarchy was assigned based on the Mouse Genome-Database (http://www.informatics.jax.org), and the interactions among the selected genes were assessed using the STRING database (http://string-db.org).

### Administration of *Lpc*-EV to mice

*Lpc*-EV was administered to mice as previously described^10,11^. *Lpc*-EV was orally administered to mice at a dose of 2.27 mg/Kg/day by drinking water from 6.5 months of age until sacrifice at 8.0 months of age. A water bottle containing *Lpc*-EV diluted in drinking water to the concentration of 15 μg/mL (1.29 × 10^9^ EV particles/mL) was presented to mice in a regular home cage. *Lpc*-EV-containing bottles were replaced every other day.

### Quantitative real-time PCR

Quantitative real-time PCR (qPCR) was carried out as previously described^22,33^. Briefly, HT22 cells cultured in a 6-well plate were harvested using TRIzol reagent (15596-018, Invitrogen). Hippocampal tissues were homogenized in TRIzol solution using pellet pestles (Z359971, Sigma‒Aldrich, Saint Louis, MO, USA), and total RNA was isolated from the homogenates. Two micrograms of total RNA were converted into cDNA using a reverse transcriptase system (Promega, Madison, WI, USA).

qPCR reaction mixture contained 4 μl of 1/8 diluted cDNA, 10 μl of 2X iQTM SYBR Green Supermix (Bio-Rad Laboratories, Foster City, CA, USA), and 1 μl each of 5 pmol/μl forward and reverse primers in 20 μl. qPCR was carried out using the CFX 96 Real-Time PCR System Detector (Bio-Rad Laboratories). Transcript levels were normalized relative to *Gapdh* and *L32* levels.

The primers used were as follows:

*Adam10*, forward 5′-AGCAACATCTGGGGACAAAC-3′ and reverse 5′-TGGCCAGATTCAACAAAAC-3′; *Apoe*, forward 5′-GCTCCCAAGTCACACAAGAA-3′ and reverse 5′-GTCGGTTGCGTAGATCCTC-3′; *Bace-1*, forward 5′-TCAAGATGGACTGCAAGGAGA-3′ and reverse 5′-AAAATGTTCCAAGGGGTCGT-3′; *Bace-2*, forward 5′-GTATAACGCAGACAAGGCCA-3′ and reverse 5′-AGAATTTGTCCAGCATGCCA-3′; *Bdnf* (total form), forward 5′-TGGCTGACACTTTTGAGCAC-3′ and reverse 5′-GTTTGCGGCATCCAGGTAAT-3′; *Cbp*, forward 5′-GGTTGCCTATGCTAAGAAAGT-3′ and reverse 5′-GATGCCTTGCTTATGTAAACG-3′; *Creb1*, forward 5′-TGGACAGCAGATTCTAGTG-3′ and reverse 5′-GGAGGACGCCATAACAAC-3′; *G9a*, forward 5′-CGCAACATCACCCATCTG-3′ and reverse 5′-TCATACCAGCATCGGATACT-3′; *Glp*, forward 5′-CCAAGCAAGAGACCAAGCAG-3′ and reverse 5′-CTTCCTGTGGGCTAGCTCTT-3′; *Hdac1*, forward 5′-CAGTGTGGCTCAGATTCCCT-3′ and reverse 5′-GGGCAGCTCATTAGGGATCT-3′; *Hdac2*, forward 5′- GGGACAGGCTTGGTTGTTTC-3′ and reverse 5′-GAGCATCAGCAATGGCAAGT-3′; *Hdac3*, forward 5′-AGAGAGGTCCCGAGGAGAAC-3′ and reverse 5′-ACTCTTGGGGACACAGCATC-3′; *Hdac4*, forward 5′-CAATCCCACAGTCTCCGTGT-3′ and reverse 5′-CAGCACCCCACTAAGGTTCA-3′; *Hdac5*, forward 5′-TGTCACCGCCAGATGTTTTG-3′ and reverse 5′-TGAGCAGAGCCGAGACACAG-3′; *Ide*, forward 5′-TTCGATGTTTCCCATGAACA-3′ and reverse 5′-ACAGGAAAAACTGCGCAAAC-3′; *Kdm4a*, forward 5′-GTCTGGCCTCTTCACTCAGT-3′ and reverse 5′- TACCATTCACGTCTGCTCCA-3′; *Kdm4b*, forward 5′-CGGGGCTTTTCACCACAGTAC-3′ and reverse 5′- GTACAGGGAGCCACTGATGT-3′; *Kdm4c*, forward 5′-GTCCCCTAAATCCCAGCTGT-3′ and reverse 5′- TAGCACTGTCTTGGCTTCCA- 3′; *Lrp1*, forward 5′-CGACATTGACGACAGGATCT-3′ and reverse 5′-CACGATCTTGCTATCCACCA-3′; *MeCP2*, forward 5′-ACAGCGGCGCTCCATTATC-3′ and reverse 5′-CCCAGTTACCGTGAAGTCAAAA-3′; *Mmp-2*, forward 5′-ATGATGATGAGCTGTGGACC-3′ and reverse 5′-GCCATACTTGCCATCCTTCT-3′; *Mmp-9*, forward 5′-AGAGGCATACTTGTACCGCT-3′ and reverse 5′-TCCCACTTGAGGCCTTTGAA-3′; *Nep*, forward 5′-GGGAGGCTTTATGTGGAAGC-3′ and reverse 5′-CCGGATTTGTGCAATCAAGT-3′; *Ngf*, forward 5′-AGCATTCCCTTGACACAG-3′ and reverse 5′-GGTCTACAGTGATGTTGC-3′; *Nt3*, forward 5′-TACTACGGCAACAGAGACG-3′ and reverse 5′-GTTGCCCACATAATCCTCC-3′; *Nt4/5*, forward 5′-AGCGTTGCCTAGGAATACAGC-3′ and reverse 5′-GGTCATGTTGGATGGGAGGTATC-3′; *p300*, forward 5′-GACTGAGCAGCGATAATG-3′ and reverse 5′-CAAGGTGTCTCTAGTGTATG-3′; *Pea3*, forward 5′-TTGTTCCTGATTTCCATTCAGA-3′ and reverse 5′-GACTCTGGGGTTCCTTCTTGA-3′; *Psen-1*, forward 5′-CGTGCTCTGCTAGCTTTGAC-3′ and reverse 5′-GCTCTGTTTGGTTCACCTCA-3′; *Psen-2*, forward 5′-GGAGAGCGAAGAAGACTGTG-3′ and reverse 5′-GCCCGTTCTTCTCAGTGTAG-3′; *Rest*, forward 5′-TGAGGGAGAGTTTGTGTGTAT-3′ and reverse 5′-AGTGGCGATTGAGGTGTT-3′; *Setdb1*, forward 5′-GGTGGTTGAAGAGCTGGGTA-3′ and reverse 5′-TCACTTCCCTGGATGCATCA-3′; *Sirt1*, forward 5′-GATCCTTCAGTGTCATGGTTC-3′ and reverse 5′-ATGGCAAGTGGCTCATCA-3′; *Sirt5*, forward 5′-ATCGCAAGGCTGGCACCAAGAA-3′ and reverse 5′-CTAAAGCTGGGC AGATCGGACT-3′; *Sirt7*, forward 5′-CTGGAGATTCCTGTCTACAACCG-3′ and reverse 5′- AGTGACTTCCTACTGTGGCTGC-3′; *SUV39H1*, forward 5′-TGGTTAAGTGGCGTGGGTAT-3′ and reverse 5′- TTGTTCCCAACGCTGAAGTG-3′; *tPa*, forward 5′-GCGAACCAAGATGCTTCAAT-3′ and reverse 5′-CTCCTTGCATTGTAGGGCTT-3′; *TrkB*, forward 5′-AAGGACTTTCATCGGGAAGCTG-3′ and reverse 5′-TCGCCCTCCACACAGACAC-3′; *Gapdh*, forward 5′-AGAAGGTGGTGAAGCAGGCATC-3′ and reverse 5′-CGAAGGTGGAAGA GTGGGAGTTG-3′; and *L32*, forward 5′-GCTGCCATCTGTTTTACGG-3′ and reverse 5′-TGACTGGTGCCTGATGAACT-3′.

### Thioflavin S staining of Aβ deposition

Thioflavin S (ThS) staining was carried out as previously described^22^. Briefly, ThS (T1892, Sigma‒Aldrich) was dissolved in 50% ethanol followed by dilution in H_2_O to 1 mM. Free-floating brain sections were washed with 1X PBS (137 mM NaCl, 2.7 mM KCl, 10 mM Na_2_HPO_4_, and 1.8 mM KH_2_PO_4_) and mounted onto a glass slide. The brain sections were incubated with 1 mM ThS for 5 min. Stained sections were washed in 100%, 95%, and 50% ethanol for 30 s each and then rinsed with 1X PBS twice. The sections were dried and cover-slipped with anti-fade fluorescent mounting medium (S3023, DAKO, Carpinteria, CA, USA). ThS-stained plaques were photographed using an Olympus BX51 microscope equipped with a DP71 camera. The number of plaques and the size of the stained area of the plaques were analyzed using MetaMorph Microscopy Automation & Image Analysis software (Molecular Devices, Sunnyvale, CA, USA).

### Immunohistochemistry

Immunocytochemical staining was carried out as previously described^22,35^. Briefly, brain sections were incubated with a primary antibody in 5% BSA solution at 4 °C overnight followed by a secondary antibody. The primary antibodies used were anti-DCX (sc-8066, Santa Cruz), anti-Ki-67 (VP-K451, Vector Laboratories), anti-MAP2 (GTX133109, Genetex), anti-Iba-1 (GTX632426, Genetex), anti-GFAP (12389, Cell Signaling), anti-MeCP2 (3456 S, Cell Signaling), and anti-Sirt1 (8469 S, Cell Signaling). A secondary antibody labeled with DyLight488 anti-rabbit (DI-1488, Vector Laboratories), DyLight594 anti-rabbit (DI-1094, Vector Laboratories), DyLight488 anti-mouse (DI-2594, Vector Laboratories), or DyLight594 anti-mouse (DI-2594, Vector Laboratories) IgG diluted at 1:200 in 1X PBST was used. For 3,3’-diaminobenzidine (DAB)-based immunohistochemistry, biotinylated goat anti-mouse IgG (BA-9200, Vector Laboratories) was used. After washing, stained signals were visualized using an ABC Elite kit (PK 6200, Vector Laboratories). Regarding the immunofluorescence staining, stained sections were mounted on a gelatin-coated slide glass with fluorescent mounting medium (S3023, DAKO, Carpinteria, CA, USA) and/or DAPI staining mounting solution (H-1200, Vector Laboratories). Stained sections and cells were analyzed using an Olympus BX 51 microscope equipped with a DP71 camera. Stained images were analyzed using MetaMorph Microscopy Automation & Image Analysis software (Molecular Devices).

### Behavioral tests

Behavioral tests were carried out as previously described^22^. The behavioral activity of mice in each test was recorded using a video-tracking system (SMART; Panlab Harvard Bioscience, Holliston, MA, USA) or a webcam recording system (HD Webcam C210, Logitech, Newark, CA, USA).

#### Novel object recognition test

The novel objective recognition test (NOR) was carried out as previously described^22^. A subject mouse was presented with two identical objects (Object A; wooden blocks; 3.5 cm × 3.5 cm × 7 cm) in the open field (40 × 40 × 35 cm) and the time spent exploring each object was recorded for 10 min. This procedure was repeated twice and regarded as the familiarization phase. Two hours after the familiarization phase, one familiar object was replaced with a new object (Object B, a 100-ml glass flask containing fresh cage bedding at a 3-cm depth; 6 cm in diameter × 10 cm in height). The time spent exploring each object was recorded for 10 min. Fifteen minutes later, the familiar object (Object A) was moved to a new location near the wall to line up with the new object (Object B) relative to the wall. Then the subject mouse was placed in the open field, and the time spent exploring each object was recorded for 10 min. This test was regarded as the novel location recognition test (NLR). Twenty-four hours after the familiarization phase, the subject mouse was presented with the familiar object (Object A) and a third, new object (Object C, a plastic block made by stacking four 60-mm culture dishes with a black-tape band; 5.5 cm in diameter × 7.5 cm in height), and the time spent with each object was recorded for 10 min.

### Water maze test

The water maze test was performed as previously described^22^. The water maze consisted of a circular pool (90 cm in diameter and 50 cm in depth) filled with water (24 °C) to a depth of 40 cm. The water was rendered opaque by the addition of nontoxic white paint. (Sargent^®^ White Art Tempera Paint). Spatial visual clues were provided with different symbols (circle, cross, oblique stripes, and checkered patterns, each with a diagonal of 40 cm) on each wall of the room. A circular platform (10 cm in diameter) was placed in the target quadrant, submerged approximately 1 cm below the surface of the water, and located in the middle of the center of the pool and the tank wall.

The acquisition phase consisted of 5 days of training trials; two trials with a 6-h interval per day. The training trial was initiated by placing the subject mouse at the start position in the pool by facing the mouse head to the wall of the tank, and the time and path taken to find the platform was recorded using a video-tracking system. A trial lasted until the mouse reached the platform or when 90 s had elapsed. If a mouse did not find the platform within 90 s, the mouse was placed on the platform for 15 s to help it acquire the context of the platform with respect to spatial cues. After the completion of each trial, the mouse was dried and returned to its home cage. This procedure was repeated 6 h later. Therefore, two trials were completed in a day. On Day 6, after the hidden platform was removed from the pool, the mouse was placed in the pool by facing the wall of the tank at the position opposite to the target quadrant and the time spent and path in the four quadrants were recorded over 60 s. Mice that did not move at a location for >30 s during the probe trial were excluded from the final data. After the probe trial test, mice were placed in the pool with the platform and a small visual flag (4.5 cm in height × 4.5 cm in width) in the target quadrant, and the time spent and path in the four quadrants were recorded over 60 sec to confirm the absence of visual or motor deficits.

#### Passive avoidance test

The passive avoidance test was carried out as previously described^22^. The test apparatus consisted of a lighted chamber (15 × 15 × 20 cm; 1500 lux) and a dark chamber (15 × 15 × 20 cm), each equipped with a metal grid floor (1 mm in diameter, 1 cm apart between grid). On the first day, a subject mouse was placed in the lighted chamber with the door opened and allowed to freely explore the equipment for 5 min. On Day 2, the mouse was placed in the light chamber. After 30 s, the middle door was opened, and the latency to enter the dark chamber was recorded, which was regarded as the pretest. When the mouse entered the dark room, the door was closed, and two successive electric foot-shocks (100 V, 0.3 mA of electrical shock delivered for 1.5 sec with a 3-second interval) were delivered through the grid floor. After shock-dark room conditioning, the mice were returned to their home cages. On Day 3 (24 h later), the mice were individually replaced in the lighted chamber, and the latency to entering the dark chamber was recorded, which was referred as the posttest. The posttest was repeated on Day 6 (72 h later) and Day 8 (120 h later). The total freezing time during the testing period on Day 3 was also analyzed.

### Statistical analysis

Two-sample comparisons were performed using Student’s *t*-test, and multiple comparisons were performed using one-way ANOVA followed by the Newman‒Keuls post hoc test, two-way ANOVA or two-way repeated-measures ANOVA followed by the Bonferroni post hoc test. All data are presented as the mean ± SEM, and statistical significance was accepted at the 5% level.

## Results

### *Lactobacillus paracasei-*derived EVs (*Lpc*-EV) counteracted Aβ42-induced downregulation of neurotrophic factors and *TrkB* in HT22 cells

**Fig. 1:**
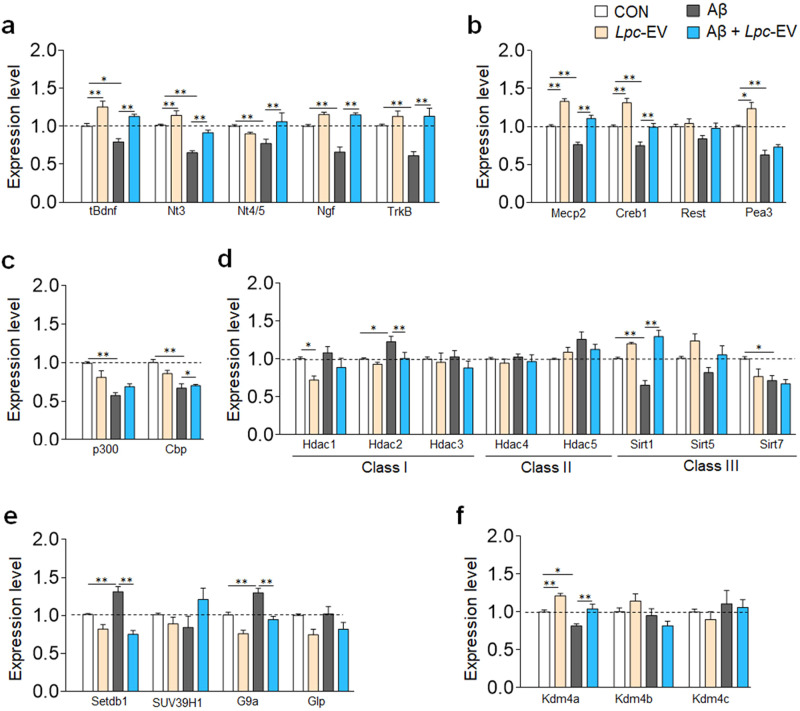
*Lpc*-EV counteracted the Aβ42-induced altered expression of neurotrophic factors and epigenetic factors in HT22 cells. **a** Expression levels of *Bdnf*, *Nt3*, *Nt4/5*, *Ngf*, and *TrkB* in HT22 cells treated with Aβ42 (1 μM, final) or Aβ42 plus *Lpc*-EV (10 μg/ml, final) for 24 h. *n* = 8 data points per group. *Bdnf*, Lpc-EV, F(1,28) = 34.94, *p* < 0.0001; Aβ42, F(1,28) = 11.10, *p* = 0.0024; interaction, F(1,28) = 0.655, *p* = 0.4253; *Nt3*, *Lpc*-EV, F(1,28) = 26.40, *p* < 0.0001; Aβ42, F(1,28) = 58.80, *p* < 0.0001; interaction, F(1,28) = 3.100, *p* = 0.0892; *Nt4/5*, *Lpc*-EV, F(1,28) = 4.432, *p* = 0.044; Aβ42, F(1,28) = 1.306, *p* = 0.2627; interaction, F(1,28) = 13.50, *p* = 0.001; *Ngf*, *Lpc*-EV, F(1,28) = 63.78, *p* < 0.0001; Aβ42, F(1,28) = 18.11, *p* = 0.002; interaction, F(1,28) = 17.15, *p* = 0.0003; and *TrkB, Lpc*-EV, F(1,28) = 20.93, *p* < 0.0001; Aβ42, F(1,28) = 7.779, *p* = 0.0094; interaction, F(1,28) = 7.918, *p* = 0.0089. **b**–**f** Expression levels of *Mecp2, Creb1, Rest*, and *Pea3* (**b**); *p300* and *Cbp* (**c**); *Hdac1*, *Hdac2*, *Hdac3*, *Hdac4*, *Hdac5*, *Sirt1*, *Sirt5*, and *Sirt7* (**d**); *Setdb1, Suv39h1, G9a*, and *Glp* (**e**); and *Kdm4a, Kdm4b*, and *Kdm4c* (**f**) in HT22 cells treated for 24 h with Aβ42 (1 μM) or Aβ42 plus *Lpc*-EV (10 μg/ml). *n* = 8 data points per group. *Mecp2, Lpc*-EV, F(1,28) = 106.6, *p* < 0.0001; Aβ42, F(1,28) = 51.84, *p* < 0.0001; interaction, F(1,28) = 0.6603, *p* = 0.8298; *Creb1, Lpc*-EV, F(1,28) = 37.21, *p* < 0.0001; Aβ42, F(1,28) = 39.40, *p* < 0.0001; interaction, F(1,28) = 0.5855, *p* = 0.4506; *Rest, Lpc*-EV, F(1,28) = 2.693, *p* = 0.1120; Aβ42, F(1,28) = 4.350, *p* = 0.0462; interaction, F(1,28) = 0.8738, *p* = 0.03579; *Pea3, Lpc*-EV, F(1,28) = 9.824, *p* = 0.0040; Aβ42, F(1,28) = 65.53, *p* < 0.0001; interaction, F(1,28) = 1.452, *p* = 0.2382; *p300*, *Lpc*-EV, F(1,28) = 0.4376, *p* = 0.5237; Aβ42, F(1,28) = 27.20, *p* < 0.0001; interaction, F(1,28) = 8.284, *p* = 0.0076; *Cbp, Lpc*-EV, F(1,28) = 2.009, *p* = 1674; Aβ42, F(1,28) = 36.91, *p* < 0.0001; interaction, F(1,28) = 4.740, *p* = 0.0381; *Hdac1*, *Lpc*-EV, F(1,28) = 8.986, *p* = 0.0056; Aβ42, F(1,28) = 2.276, *p* = 0.1426; interaction, F(1,28) = 0.3226, *p* = 0.5746; *Hdac2*, *Lpc*-EV, F(1,28) = 5.148, *p* = 0.0312; Aβ42, F(1,28) = 8.124, *p* = 0.0081; interaction, F(1,28) = 1.210, *p* = 0.2807; *Hdac3*, *Lpc*-EV, F(1,28) = 1.307, *p* = 0.2626; Aβ42, F(1,28) = 0.1064, *p* = 0.7468; interaction, F(1,28) = 0.2668, *p* = 0.6095; *Hdac4*, *Lpc*-EV, F(1,28) = 0.9985, *p* = 0.3262; Aβ42, F(1,28) = 0.1629, *p* = 0.6895; interaction, F(1,28) = 0.0017, *p* = 0.9674; *Hdac5*, *Lpc*-EV, F(1,28) = 0.0856, *p* = 0.7720; Aβ42, F(1,28) = 4.909, *p* = 0.0350; interaction, F(1,28) = 2.671, *p* = 0.1134; *Sirt1*, *Lpc*-EV, F(1,28) = 63.93, *p* < 0.0001; Aβ42, F(1,28) = 5.902, *p* = 0.0218; interaction, F(1,28) = 18.12, *p* = 0.0002; *Sirt5, Lpc*-EV, F(1,28) = 8.020, *p* = 0.0085; Aβ42, F(1,28) = 5.055, *p* = 0.0326; interaction, F(1,28) = 0.0014, *p* = 0.9703; *Sirt7, Lpc*-EV, F(1,28) = 5.081, *p* = 0.0322; Aβ42, F(1,28) = 9.163, *p* = 0.0053; interaction, F(1,28) = 2.602, *p* = 0.1179; *Setdb1, Lpc*-EV, F(1,28) = 52.37, *p* < 0.0001; Aβ42, F(1,28) = 4.838, *p* = 0.0363; interaction, F(1,28) = 12.53, *p* = 0.0.0014; *Suv39h1, Lpc*-EV, F(1,28) = 40.00, *p* < 0.0001; Aβ42, F(1,28) = 9.665, *p* = 0.0043; interaction, F(1,28) = 3.643, *p* = 0.0666; *G9a, Lpc*-EV, F(1,28) = 41.90, *p* < 0.0001; Aβ42, F(1,28) = 26.68, *p* < 0.0001; interaction, F(1,28) = 1.454, *p* = 0.2380; *Glp, Lpc*-EV, F(1,28) = 9.102, *p* = 0.0054; Aβ42, F(1,28) = 0.3600, *p* = 0.5533; interaction, F(1,28) = 1.1048, *p* = 0.7486; *Kdm4a, Lpc*-EV, F(1,28) = 28.80, *p* < 0.0001; Aβ42, F(1,28) = 19.34, *p* = 0.0001; interaction, F(1,28) = 0.0258, *p* = 0.8736; *Kdm4b, Lpc*-EV, F(1,28) = 0.0023, *p* = 0.9619; Aβ42, F(1,28) = 6.015, *p* = 0.0207; interaction, F(1,28) = 3.211, *p* = 0.0840; and *Kdm4c, Lpc*-EV, F(1,28) = 0.4177, *p* = 0.5234; Aβ42, F(1,28) = 1.319, *p* = 0.2604; interaction, F(1,28) = 0.0496, *p* = 0.8254. Data are presented as the mean ± SEM. *p < 0.05; **p < 0.01 (two-way ANOVA followed by the Bonferroni post hoc test).

Recently we reported that *Lactobacillus plantarum-*derived EVs has the ability to upregulate the expression of neurotrophic factors and *TrkB* in HT22 neuronal cells. We investigated whether *Lpc*-EV produces similar effects in HT22 neuronal cells that have been treated with Aβ42. Treatment with Aβ42 in HT22 cells downregulated the expression of *Bdnf, Nt3, Nt4/5*, *Ngf*, and *TrkB*. In contrast, treatment with *Lpc*-EV blocked the Aβ42-induced downregulation of *Bdnf, Nt3, Nt4/5, Ngf*, and *TrkB* (Fig. 1a).

We used two independent approaches to identify genetic or epigenetic factors that regulate the transcriptional effects of Aβ42 and *Lpc*-EV on the neurotrophin system. First, we investigated whether any known nuclear factors mediate the reversing effects of *Lpc*-EV on the expression of neurotrophic factors. A number of genetic and epigenetic factors, including Creb1, Rest, and Pea3^38–40^, and MeCP2 and Sirt1^19,20,22,26,33,38,41,42^ have been studied for their role in regulating the expression of neurotrophic factors. Therefore, we selected a number of gene sets that included those characterized to regulate the expression of neurotrophic factors in previous studies, and included genes that are involved in the acetylation, deacetylation, methylation and demethylation of histones (ex, histone H3K9), which are important in regulating neuronal plasticity in various pathologies^10,19,26,33,38,40^. Aβ42 treatment in HT22 cells downregulated the expression of *Mecp2, Creb1*, and *Pea3*. In contrast, treatment with *Lpc*-EV blocked Aβ42-induced downregulation of *Mecp2* and *Creb1* but not *Rest* or *Pea3* (Fig. 1b). We investigated whether Aβ42 and *Lpc*-EV treatment affect the expression of histone modification factors. Aβ42 downregulated the expression of the histone acetyltransferases *p300* and *CBP*; the histone deacetylases (HDAC) *Sirt1, Sirt5*, and *Sirt7*; and the histone-lysine demethylase *Kdm4a*. *Lpc*-EV upregulated the expression of *Hdac2* and the histone-lysine methyltransferases *Setdb1* and *G9a*. In contrast, *Lpc*-EV blocked the Aβ42-induced downregulation of *Sirt1, Sirt5*, and *Kdm4a*, and Aβ42-induced upregulation of *Hdac2*, *Setdb1*, and *G9a* (Fig. 1c–f). These results suggest that *Lpc*-EV has the ability to induce transcriptional responses of multiple epigenetic factors including *Mecp2* and *Sirt1*.

**Fig. 2:**
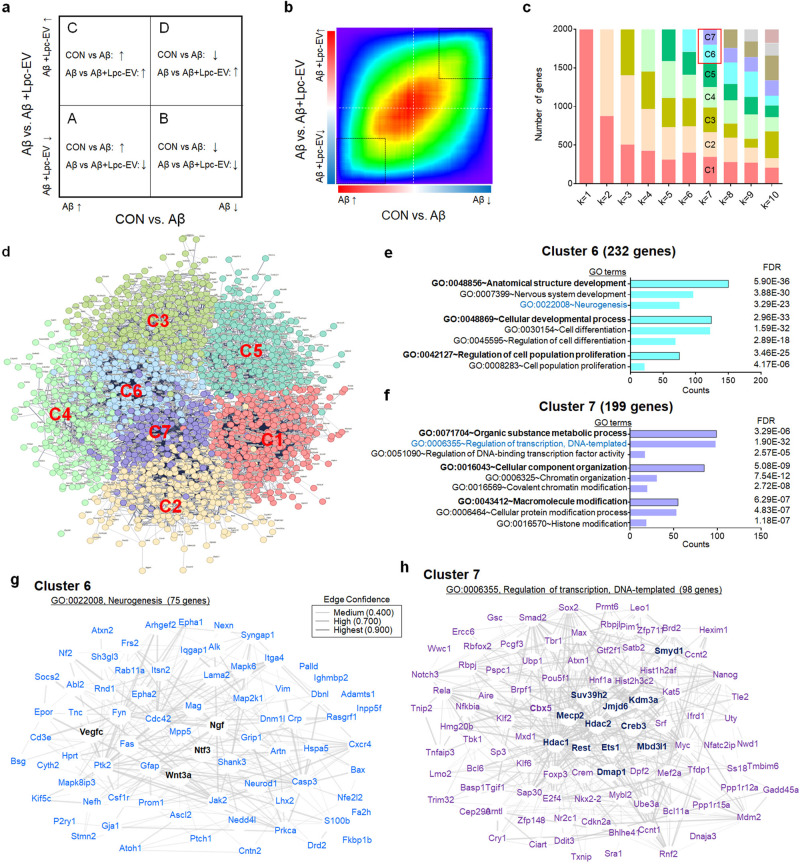
Genome-wide microarray analysis identified a list of genes that were differentially expressed by Aβ42 and their altered expression was reversed by Lpc-EV. **a**, **b** A hypothetical RROH map with up- or downregulated genes by Aβ42 or *Lpc*-EV (**a**). A hypergeometric map between the genes changed by Aβ42 (*x*-axis) and those by *Lpc*-EV (*y*-axis). The genes were ranked based on expression differences between two comparison groups by their log_10_-transformed *t*-test *p* values and plotted on the *x*- and *y*-axes (**b**). Quadrants A and D contained 11,003 and 9,479 transcripts, respectively. The top 20% of transcripts ranked on expression differences were selected and used for further analyses. Up and down arrows indicate upregulated and downregulated expression, respectively. **c**, **d** Serial K-means clustering of the selected genes and subsequent Gene Ontology (GO) enrichment analyses led to identify seven functional clusters (**c**), which were visualized by a combined PPI network (**d**). Clusters 6 and 7 contained functional groups of genes for neurotrophic factors (Cluster 6) and for transcription and epigenetic regulation (Cluster 7). **e**, **h** A summary of key features of Clusters 6 (**e**, **g**) and 7 (**f**, **h**). Top three functional groups assigned by GO terms in Cluster 6 (**e**) and the PPI of 75 genes assigned with a GO term for “neurogenesis” (**g**). Top three functional groups covered by GO terms in Cluster 7 (**f**) and the PPI of 98 genes assigned with a GO term for “regulation of transcription, DNA-templated” (**h**).

Second, we investigated the effects of *Lpc*-EV on the transcriptional responses of genes induced by Aβ42 using a genome-wide approach. We used a microarray assay followed by Rank-Rank Hypergeometric Overlap (RRHO)^36,37^ to identify differentially regulated genes after Aβ42 treatment and their changes were reversed by *Lpc*-EV. Of the 32,317 transcripts in the microarray, 11,003 transcripts (34%) were upregulated, and the altered expression of those transcripts was reversed by *Lpc*-EV treatment (Quadrant A). In addition, 9479 transcripts (29%) were downregulated by Aβ42 and their altered expression was reversed by *Lpc*-EV (Quadrant D) (Fig. 2a, b). The transcripts in Quadrants A and D (11,003 and 9479, respectively) were first annotated with *Mus musculus* genes, and then the top 20% of transcripts by rank based on expression differences were selected. This resulted in 1321 genes in Quadrant A and 1079 genes in Quadrant D. Finally, the transcripts with a high confidence interaction score of 0.7 or higher in the STRING database were selected. This resulted in 1197 genes in Quadrant A and 799 genes in Quadrant D (Fig. 2a, b).

Serial *K*-means clustering and Gene Ontology (GO) enrichment analyses identified seven functional clusters of genes (Fig. 2c, d). The identified functional clusters had the following features (Fig. 2e, f; Supplementary Fig. 1). Cluster #6 contained genes covered by GO terms for “neurogenesis”, which included *Nt3, Ngf, Vegfc*, and *Wnt3a* (Fig. 2e, g). Cluster #7 contained genes assigned by GO terms for “regulation of transcription, DNA-templated”, “chromatin organization”, and “histone modification”, which included *Mecp2*; *Hdac1* and *Hdac2* (histone deacetylases); *Suv39h2* and *Smyd1* (histone methyltransferases); *Kdm3a* and *Jmjd6* (histone demethylases), *Rest, Ets1*, and *Creb3* (transcription factors involved in the expression of *Bdnf, Vegf* and *MMPs*); and *Mbd3l1* and *Dmap1* (methylated DNA related factors) (Fig. 2e–h). Functional protein‒protein interactions (PPI) constructed for genes assigned by GO terms for “neurogenesis” and for “regulation of transcription, DNA-templated” showed intimate interactions with high interaction confidence scores among the identified genes (Fig. 2g, h).

Cluster #1 contained genes involved in “Organic substance metabolic process”, which included factors involved in RNA processing, and “Cellular component organization or biogenesis”; Cluster #2 contained genes involved in “Organic substance metabolic process”, which included factors involved in DNA metabolic process, and “Cell cycle”; Cluster #3 contained genes involved in “Establishment of localization”, which included factors involved in ion transport, and “Homeostatic process”; Cluster #4 contained genes involved in “Regulation of biological process”, which included factors involved in regulation of signaling, and “Cell communication”; and Cluster #5 contained genes involved in “Cellular metabolic process”, which included factors involved in carboxylic acid metabolic process, and “Nitrogen compound metabolic process” (Supplementary Fig. 1). These results suggest that *Lpc*-EV can block the changes in cellular and transcriptional responses that are induced by Aβ42.

### *Lpc*-EV-induced upregulation of neurotrophic factors and *TrkB* in HT22 cells was mediated by epigenetic factors

**Fig. 3:**
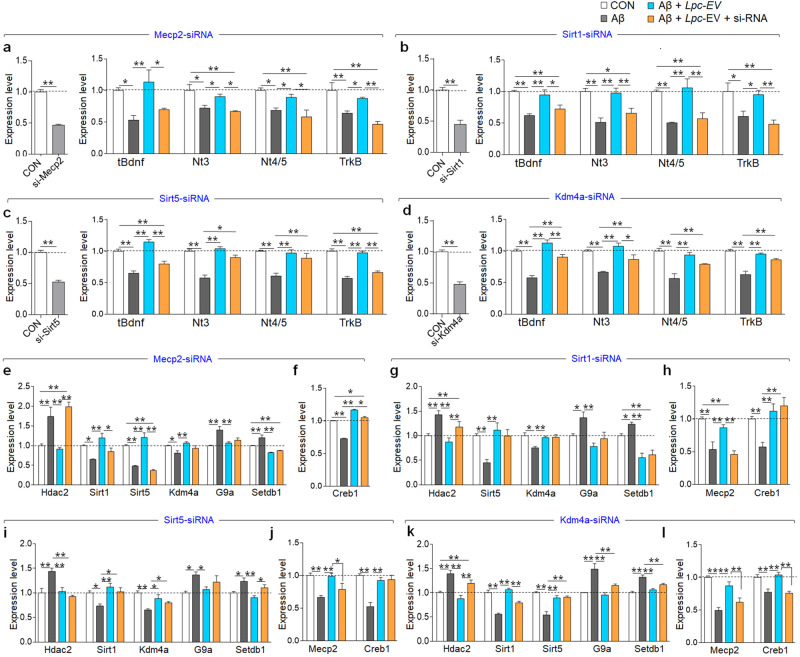
*Lpc*-EV counteracted the Aβ42-induced downregulation of neurotrophic factors via the upregulation of epigenetic factors in HT22 cells. **a**–**d** Expression levels of *Bdnf*, *Nt3*, *Nt4/5*, and *TrkB* in HT22 cells treated with Aβ42 (1 μM), Aβ42 plus *Lpc*-EV (10 μg/ml), or Aβ42 plus *Lpc*-EV and the indicated siRNA: siRNA-Mecp2 (**a**), siRNA-Sirt1 (**b**), siRNA-Sirt5 (**c**), and siRNA-Kdm4a (**d**). siRNA-control, siCON. *n* = 8 per group. siRNA-Mecp2 (**a**), t(14) = 19.57, *p* < 0.0001; t*Bdnf*, F(3,28) = 6.796, *p* = 0.0063; *Nt3*, F(3,28) = 7.576, *p* = 0.0042; *Nt4/5*, F(3,28) = 8.263, *p* = 0.0030; *TrkB*, F(3,28) = 12.02, *p* = 0.0006. siRNA-Sirt1 (**b**), t(14) = 10.80, *p* < 0.0001; t*Bdnf*, F(3,28) = 11.67, *p* = 0.0007; *Nt3*, F(3,28) = 12.06, *p* = 0.0006; *Nt4/5*, F(3,28) = 11.13, *p* = 0.0009; *TrkB*, F(3,28) = 7.572, *p* = 0.0042. siRNA-Sirt5 (**c**), t(14) = 13.15, *p* < 0.0001; t*Bdnf*, F(3,28) = 34.05, *p* < 0.0001; *Nt3*, F(3,28) = 30.44, *p* < 0.0001; *Nt4/5*, F(3,28) = 12.35, *p* = 0.0002; *TrkB*, F(3,28) = 56.56, *p* < 0.0001. siRNA-Kdm4a (**d**), t(14) = 19.51, *p* < 0.0001; t*Bdnf*, F(3,28) = 35.21, *p* < 0.0001; *Nt3*, F(3,28) = 15.21, *p* = 0.0002; *Nt4/5*, F(3,28) = 16.18, *p* = 0.0002; *TrkB*, F(3,28) = 26.28, *p* < 0.0001. **e**–**l** Expression of *Hdac2, Sirt1, Sirt5, Setdb1, Kdm4a, G9a*, *Mecp2*, and *Creb1* in HT22 cells treated with Aβ42 (1 μM), Aβ42 plus *Lpc*-EV (10 μg/ml), or Aβ42 and *Lpc*-EV plus the indicated siRNA: siRNA-MeCP2 (**e**, **f**), siRNA-Sirt1 (**g**, **h**), siRNA-Sirt5 (**i**, **j**), and siRNA-Kdm4a (**k**, **l**). *n* = 8 per group. siRNA-MeCP2 (**e**, **f**); *Hdac2*, F(3, 28) = 15.57, *p* = 0.0002; *Sirt1*, F(3,28) = 8.921, *p* = 0.0022; *Sirt5*, F(3,28) = 42.61, *p* < 0.0001; *Kdm4a*, F(3,28) = 5.852, *p* = 0.0106; *G9a*, F(3,28) = 10.03, *p* = 0.0014; *Setdb1*, F(3,28) = 92.29, *p* < 0.0001; *Creb1*, F(3,28) = 251.8, *p* < 0.0001. siRNA-Sirt1 (**g**, **h**); *Hdac2*, F(3,28) = 8.184, *p* = 0.0031; *Sirt5*, F(3,28) = 8.068, *p* = 0.0033; *Kdm4a*, F(3,28) = 6.260, *p* = 0.0084; *G9a*, F(3,28) = 6.769, *p* = 0.0064; *Setdb1*, F(3,28) = 19.82, *p* < 0.0001; *Mecp2*, F(3,28) = 13.39, *p* = 0.0004; *Creb1*, F(3,28) = 8.748, *p* = 0.0024. siRNA-Sirt5 (**i**, **j**); *Hdac2*, F(3,28) = 10.66, *p* = 0.0011; *Sirt1*, F(3,28) = 6.476, *p* = 0.0074; *Kdm4a*, F(3,28) = 9.403, *p* = 0.0018; *G9a*, F(3,28) = 4.318, *p* = 0.0278; *Setdb1*, F(3,28) = 7.358, *p* = 0.0047; *Mecp2*, F(3,28) = 8.037, *p* = 0.0010; *Creb1*, F(3,28) = 17.04, *p* < 0.0001. siRNA-Kdm4a (**k**, **l**); *Hdac2*, F(3,28) = 14.64, *p* = 0.0003; *Sirt1*, F(3,28) = 42.24, *p* < 0.0001; *Sirt5*, F(3,28) = 19.79, *p* < 0.0001; *G9a*, F(3,28) = 14.07, *p* = 0.0003; *Setdb1*, F(3,28) = 18.06, *p* < 0.0001. Data are presented as the mean ± SEM. *p < 0.05; **p < 0.01 (Student’s *t*-test; and one-way ANOVA followed by the Newman‒Keuls post hoc test).

Our initial investigation (Fig. 1) and genome-wide analysis (Fig. 2; Supplementary Fig. 1) showed that a number of genes with potentially diverse biological functions are involved in regulating Aβ42-induced changes and the effects of *Lpc*-EV. Of those identified factors, in this study, we focused on and investigated the changes in the expression of neurotrophic factors by Aβ42 and *Lpc*-EV and their potential upstream regulators. siRNA-mediated *Mecp2* knockdown in HT22 cells blocked *Lpc*-EV-induced upregulation of *Bdnf*, *Nt3*, *Nt4/5*, and *TrkB*. siRNA-mediated *Sirt1* knockdown produced similar effects (Fig. 3a, b). siRNA-mediated *Sirt5* knockdown blocked *Lpc*-EV-induced upregulation of *Bdnf* and *TrkB* but not *Nt3* or *Nt4/5* (Fig. 3c). siRNA-mediated *Kdm4a* knockdown weakly blocked the *Lpc*-EV-induced upregulated expression of *Bdnf* and *Nt3* but not *Nt4/5* or *TrkB* (Fig. 3d).

siRNA-mediated *Hdac2* knockdown partially blocked the Aβ42-induced *Bdnf*, *Nt4/5*, and *TrkB* downregulation but did not affect *Nt3* expression. *Hdac2* knockdown tended to enhance the *Lpc*-EV-induced upregulation of *Bdnf* and *Nt4/5* but did not affect *Nt3* or *TrkB* expression (Supplementary Fig. 2a). siRNA-mediated *Setdb1* knockdown produced no effect on the Aβ42-induced downregulation of *Bdnf*, *Nt3*, *Nt4/5*, and *TrkB*, and it also produced no significant effect on the *Lpc*-EV-induced upregulation of these genes (Supplementary Fig. 2b). siRNA-mediated *G9a* knockdown blocked the Aβ42-induced downregulation of *Bdnf* and *TrkB*, but did not affect *Nt3* and *Nt4/5* expression. *G9a* knockdown partially suppressed the *Lpc*-EV-induced upregulation of *Nt4/5*, but it exerted no significant effect on the *Lpc*-EV-induced upregulation of *Bdnf*, *Nt3*, and *TrkB* (Supplementary Fig. 2c).

Overall, these results suggest that *Lpc*-EV induces the upregulation of *Bdnf*, *Nt3*, *Nt4/5*, and *TrkB* by transcriptionally activating or suppressing multiple epigenetic factors. Of these, *MeCP2* and *Sirt1* serve as common mediators for *Lpc*-EV-induced effects on the expression of these genes, whereas *Sirt5, Kdm4a, Hdac2*, and *G9a* are selectively involved in the regulation of a subset of the genes.

### *Lpc*-EV-induced transcriptional activation was mediated by the upregulation of multiple epigenetic factors

Next, we investigated the hierarchical relationship among the identified epigenetic factors using a molecular genetics method. siRNA-mediated *Mecp2* knockdown blocked *Lpc*-EV-induced restoration of *Hdac2, Sirt1*, and *Sirt5* expression, and weakly blocked *Creb1* expression, but its knockdown produced no significant effect on *Kdm4a, G9a*, and *Setdb1* (Fig. 3e, f). Conversely, *Mecp2* overexpression blocked the Aβ-induced downregulation of *Sirt1, tBdnf*, and *TrkB* (Supplementary Fig. 3). siRNA-mediated *Sirt1* knockdown blocked the *Lpc*-EV-induced restoration of *Hdac2* and *Mecp2* expression, but *Sirt1* knockdown produced no significant effect on *Sirt5*, *Kdm4a, G9a, Setdb1*, and *Creb1* (Fig. 3g, h). siRNA-mediated *Sirt5* knockdown blocked the *Lpc*-EV-induced changes in *Setdb1* and *Mecp2*, but its knockdown produced no or insignificant effect on *Hdac2, Sirt1*, *Kdm4a, G9a*, and *Creb1* (Fig. 3i, j). siRNA-mediated *Kdm4a* knockdown significantly blocked the *Lpc*-EV-induced restoration of *Hdac2, Sirt1, Mecp2*, and *Creb1* expression, but *Kdm4a* knockdown produced no significant effect on *Sirt5, G9a*, and *Setdb1* (Fig. 3k, l).

siRNA-mediated *Hdac2* knockdown tended to block the *Lpc*-EV-induced upregulation of *Sirt1* and *Sirt5*, but its knockdown produced no significant effect on the *Lpc*-EV-induced changes in the expression of *Kdm4a, G9a, Setdb1, Mecp2*, and *Creb1* (Supplementary Fig. 2d, e). siRNA-mediated *Setdb1* knockdown partially enhanced the *Lpc*-EV-induced suppression of *G9a* expression, but its knockdown produced no significant effect on *Lpc*-EV-induced changes in *Hdac2, Sirt1, Sirt5*, *Kdm4a, Mecp2*, and *Creb1* (Supplementary Fig. 2f, g). siRNA-mediated *G9a* knockdown partially suppressed the *Lpc*-EV-induced upregulation of *Sirt1*, but its knockdown produced no significant effect on the *Lpc*-EV-induced changes in *Hdac2, Sirt5*, *Kdm4a, Setdb1, Mecp2*, and *Creb1* (Supplementary Fig. 2h, i).

Overall, these results suggest that the *Lpc*-EV-induced upregulation of *Bdnf, Nt3, Nt4/5*, and *TrkB* is mediated by activation of multiple epigenetic factors that interact with each other, while partly converging onto *Mecp2* and *Sirt1*.

### *Lpc*-EV treatment restored the downregulated expression of neurotrophic factors in the hippocampus of Tg-APP/PS1 mice

**Fig. 4:**
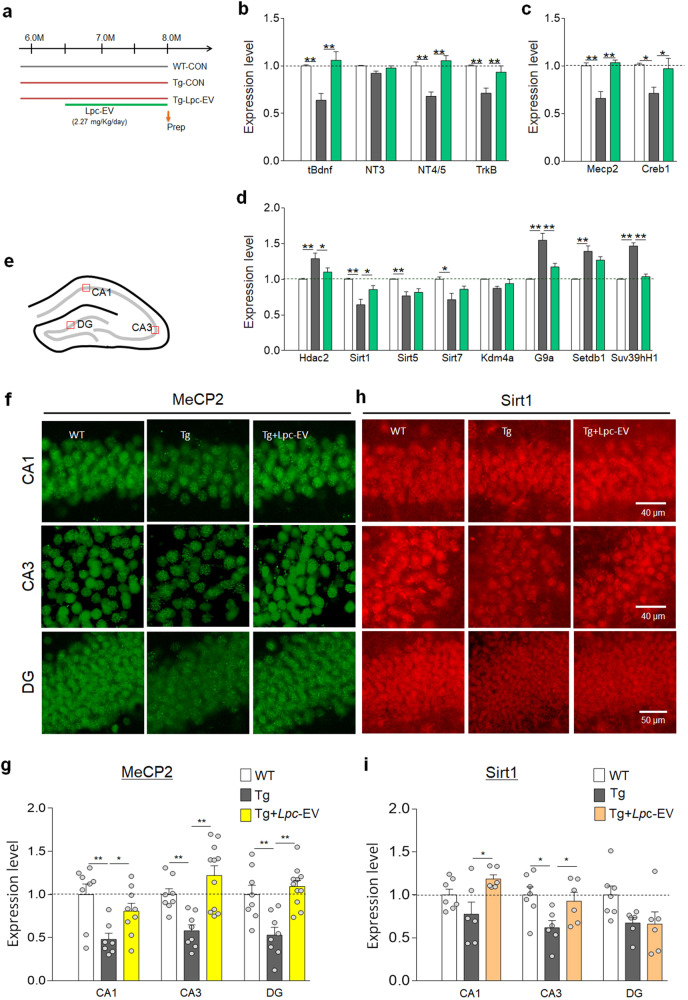
*Lpc*-EV treatment restored the expression of neurotrophic factors and epigenetic factors in Tg-APP/PS1 mice. **a** Experimental design. Tg-APP/PS1 mice were orally administered *Lpc*-EV at a dose of 2.27 mg/kg/day from 6.5 months of age until sacrifice at 8 months. Arrow, time point for tissue preparation. **b**–**d** Expression levels of *Bdnf*, *Nt3*, *Nt4/5*, and *TrkB* (**b**); *Mecp2*, and *Creb1* (**c**); *Hdac2, Sirt1, Sirt5, Sirt7, Kdm4a, G9a, Setdb1*, and *Suv39h1* (**d**) in the hippocampus of wild-type control (WT), Tg-APP/PS1 mice (Tg), and Tg-APP/PS1 mice treated with *Lpc*-EV (Tg+ *Lpc*-EV). *N* = 6 per group. *tBdnf*, F(2,5) = 1.41, *p* = 0.0010; *Nt3*, F(2,15) = 0.6496, *p* = 0.5363; *Nt4/5*, F(2,15) = 8.35, *p* < 0.0001; *TrkB*, F(2,15) = 0.977, *p* = 0.0027; *Mecp2*, F(2,15) = 19.26, *p* < 0.0001; *Creb1*, F(2,15) = 4.858, *p* = 0.0236; *Hdac2*, F(2,15) = 6.694, *p* = 0.0084; *Sirt1*, F(2,21) = 10.73, *p* = 0.0006; *Sirt5*, F(2,15) = 7.347, *p* = 0.0060; *Sirt7*, F(2,15) = 5.725, *p* = 0.0142; *Kdm4a*, F(2,15) = 2.872, *p* = 0.0879; *G9a*, F(2,15) = 19.53, *p* < 0.0001; *Setdb1*, F(2,15) = 15.09, *p* = 0.0003; *Suv39h1*, F(2,15) = 59.31, *p* < 0.0001. **e**–**i** A diagram of the hippocampus and the regions examined (**e**). Photomicrographs showing MeCP2 (**f**) and Sirt1 (**h**) expression in CA1 and CA3 pyramidal neurons and DG neurons and their quantification levels (**g**, **i**) in wild-type control (WT), Tg-APP/PS1 mice (Tg-CON), and Tg-APP/PS1 mice treated with *Lpc*-EV (Tg+*Lpc*-EV). Scale bar, 100 μm. *n* = 7–11 animals. *Mecp2*; CA1, F(2,21) = 6.504, *p* = 0.0063; CA3, F(2,24) = 11.88, *p* = 0.0003; DG, F(2,24) = 11.83, *p* = 0.0003. *n* = 6–7 animals. *Sirt1*; CA1, F(2,16) = 4.694, *p* = 0.0249; CA3, F(2,16) = 4.605, *p* = 0.0263; DG, F(2,16) = 3.377, *p* = 0.0598. Data are presented as the mean ± SEM. *p < 0.05; **p < 0.01 (one-way ANOVA followed by the Newman‒Keuls post hoc test).

We investigated whether *Lpc*-EV administration could change the expression of neurotrophic factors and *TrkB* in the brains of Tg-APP/PS1 mice. Compared to wild-type mice, Tg-APP/PS1 mice at 8 months of age had significantly downregulated expression of *Bdnf*, *Nt4/5*, and *TrkB* in the hippocampus. In contrast, Tg-APP/PS1 mice treated with *Lpc*-EV had upregulated expression of *Bdnf*, *Nt4/5*, and *TrkB* compared to that in wild-type mice (Fig. 4a, b).

Compared to wild-type mice, Tg-APP/PS1 mice also showed downregulated expression of *Mecp2, Creb1 Sirt1, Sirt5*, and *Sirt7* and upregulated expression of *Hdac2, G9a, Setdb1*, and *Suv39h1* in the hippocampus. In contrast, Tg-APP/PS1 mice treated with *Lpc*-EV had significantly upregulated expression of *Mecp2, Creb1* and *Sirt1*, and downregulated expression of *Hdac2, G9a*, and *Suv39h1* in the hippocampus (Fig. 4c, d).

Immunohistochemical analysis indicated that compared to wild-type mice, Tg-APP/PS1 mice exhibited downregulated expression of MeCP2 and Sirt1 in pyramidal and granule neurons of the hippocampus. In contrast, Tg-APP/PS1 mice treated with *Lpc*-EV showed upregulated expression of MeCP2 and Sirt1 in the hippocampus compared to that in the hippocampus of Tg-APP/PS1 mice (Fig. 4e–i), suggesting that *Lpc*-EV treatment restored the downregulated expression of MeCP2 and Sirt1 proteins in the hippocampal neuronal neurons of Tg-APP/PS1 mice.

### *Lpc*-EV treatment rescued the downregulated expression of *Mmp-2, Mmp-9*, and *Nep*, and alleviated Aβ plaque accumulation in the brains of Tg-APP/PS1 mice

**Fig. 5:**
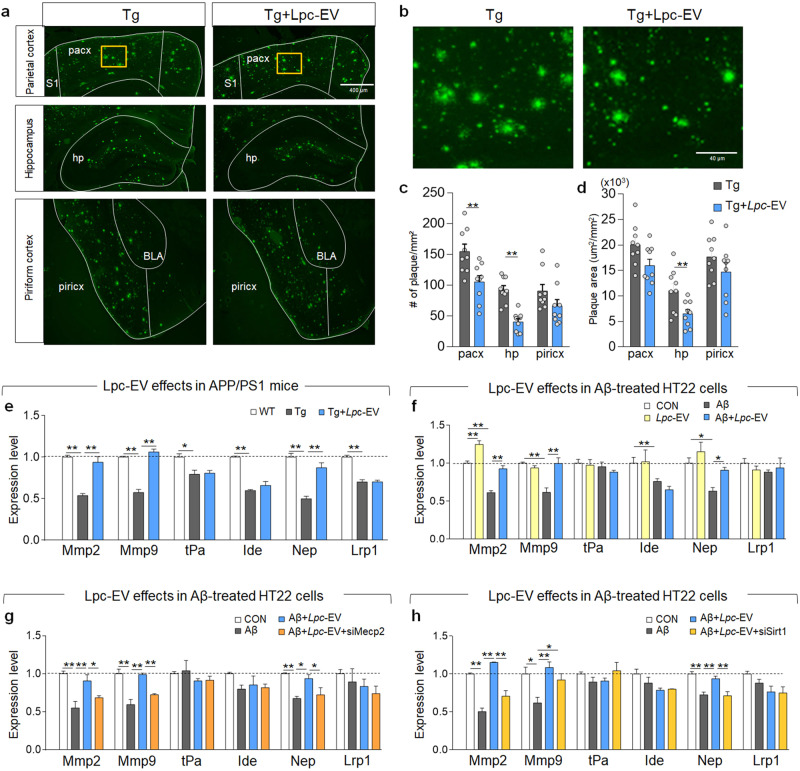
*Lpc*-EV treatment restored the Aβ42-induced downregulation of Aβ-degrading enzymes, and suppressed Aβ accumulation in Tg-APP/PS1 mice. **a**, **b** Photomicrographs showing the thioflavin S-stained parietal cortex, hippocampus, and piriform cortex (**a**) of Tg-APP/PS1 mice, and Tg-APP/PS1 mice treated with *Lpc*-EV at 8 months of age. Higher magnification of the boxed areas in the parietal cortex (**b**). *Lpc*-EV was orally administered at a dose of 2.27 mg/kg/day, as depicted in Fig. 3A. **c**, **d** Quantification of the number of plaques (**c**) and total plaque area (**d**) in the parietal cortex, hippocampus, and piriform cortex of Tg-APP/PS1 mice, and Tg-APP/PS1 mice treated with *Lpc*-EV. *n* = 9 animals per group. Plaque numbers: CA1, t(16) = 3.122, *p* = 0.0066; CA3, t(16) = 5.968, *p* < 0.0001; DG, t(16) = 1.597, *p* = 0.1298. Plaque area; CA1, t(16) = 2.016, *p* = 0.061; CA3, t(16) = 3.034, *p* = 0.0079; DG, t(16) = 1.300, *p* = 0.2121. **e** Expression levels of *Mmp-2, Mmp-9, tPA, Ide, Nep*, and *Lrp1* in the hippocampi of WT mice, Tg-APP/PS1 mice, and Tg-APP/PS1 mice treated with *Lpc*-EV. *n* = 8 animals per group. *Mmp-2*, F(2,21) = 43.29; *p* < 0.0001; *Mmp-9*, F(2,21) = 67.69, *p* < 0.0001; *tPa*, F(2,21) = 7.643, *p* = 0.0115; *Ide*, F(2,21) = 37.29, *p* < 0.0001; *Nep*, F(2,21) = 38.69, *p* < 0.0001; *Lrp1*, F(2,21) = 44.16, *p* < 0.0001. **f** Expression levels of *Mmp-2, Mmp-9, uPA, Ide, Nep*, and *Lrp1* in HT22 cells (CON), HT22 cells treated with *Lpc*-EV (10 μg/ml), HT22 cells treated with Aβ42 (1 μM), and HT22 cells treated with Aβ42 plus *Lpc*-EV. *n* = 8 per group. *Mmp-2*, F(3,28) = 49.54, *p* < 0.0001; *Mmp-9*, F(3,28) = 13.13, *p* = 0.0004; *tPa*, F(3,28) = 0.9267, *p* = 0.4505; *Ide*, F(3,28) = 4.003, *p* = 0.0345; *Nep*, F(3,28) = 7.959, *p* = 0.0035; *Lrp1*, F(3,28) = 0.4260, *p* = 0.7380. **g**, **h** Expression levels of *Mmp-2, Mmp-9, uPA, Ide, Nep*, and *Lrp1* in HT22 cells (CON), HT22 cells treated with Aβ42, and HT22 cells treated with Aβ42 plus the indicated siRNA; *siRNA-*MeCP2 (**g**) and *siRNA-*Sirt1 (**h**). *n* = 8 per group. *siRNA-*MeCP2*; Mmp-2*, F(3,28) = 10.49, *p* = 0.0011; *Mmp-9*, F(3,28) = 17.91, *p* < 0.0001; *tPa*, F(3,28) = 0.7474, *p* = 0.5444; *Ide*, F(3,28) = 1.777, *p* = 0.2050; *Nep*, F(3,28) = 8.157, *p* = 0.0032; *Lrp1*, F(3,28) = 0.9080, *p* = 0.4659. *siRNA-*Sirt1; *Mmp-2*, F(3,28) = 45.19, *p* < 0.0001; *Mmp-9*, F(3,28) = 6.416; *p* = 0.0077; *tPa*, F(3,28) = 1.127, *p* = 0.3619; *Ide*, F(3,28) = 3.498, *p* = 0.0497; *Nep*, F(3,28) = 12.95, *p* = 0.0005; *Lrp1*, F(3,28) = 3.338, *p* = 0.0561. Data are presented as the mean ± SEM. *p < 0.05; **p < 0.01 (Student’s *t*-test; one-way ANOVA followed by the Newman‒Keuls post hoc test; and two-way ANOVA followed by the Bonferroni post hoc test).

Thioflavin-S (ThS) staining indicated that *Lpc*-EV treatment tended to reduce the number and total area of ThS-stained plaques in the parietal cortex and hippocampus of Tg-APP/PS1 mice compared to Tg-APP/PS1 mice (Fig. 5a–d).

We explored the cellular factors known to regulate Aβ production and Aβ clearance. The expression levels of *Presenilin-1 (Psen1)* and *Psen2* in the hippocampus of Tg-APP/PS1 mice were slightly enhanced compared to those in wild-type mice, whereas the expression levels of *Beta-secretase 1 (Bace1), Bace2, A Disintegrin and Metallopeptidase Domain 10 (Adam10)* and *Apolipoprotein E (Apoe)* were not significantly changed. Tg-APP/PS1 mice treated with *Lpc*-EV had similar expression levels of those factors to Tg-APP/PS1 mice (Supplementary Fig. 4a). The expression levels of *Matrix metalloproteinases-2 (Mmp-2), Mmp-9*, *tissue plasminogen activator (tPA), Insulin-degrading enzyme (Ide), Neprilysin (Nep)*, and *Low-density lipoprotein receptor–related protein-1 (Lrp1)* in the hippocampus of Tg-APP/PS1 mice were reduced compared to those in wild-type mice. In contrast, compared to Tg-APP/PS1 mice, Tg-APP/PS1 mice treated with *Lpc*-EV had significantly increased expression levels of *Mmp-2*, *Mmp-9*, and *Nep* but not *tPA, Ide*, or *Lrp* (Fig. 5e).

Aβ42 treatment in HT22 cells tended to upregulate the expression of *Bace1*, *Psen1*, *Psen2, Adam10*, and *Apoe*. *Lpc*-EV treatment did not significantly change the Aβ42-induced expression of those genes (Supplementary Fig. 4b). Aβ42 treatment in HT22 cells downregulated the expression of *Mmp-2*, *Mmp-9*, uPA, and *Nep*, but not *Ide* and *Lrp*. In contrast, *Lpc*-EV treatment blocked the Aβ42-induced downregulation of *Mmp-2*, *Mmp-9*, and *Nep* (Fig. 5f).

Next, we tested whether the *Lpc*-EV-induced upregulation of *Mmp-2, Mmp-9*, and *Nep* is MeCP2- or Sirt1-dependent. siRNA-mediated *Mecp2* knockdown in HT22 cells did not affect the *Lpc*-EV-dependent changes in the expression of *Bace1, Psen1, Psen2, Adam10, Apoe*, *tPA, Ide*, and *Lrp1*. In contrast, *Mecp2* knockdown blocked the *Lpc*-EV-induced upregulation of *Mmp-2, Mmp-9* and *Nep* (Fig. 5g; Supplementary Fig. 4c). siRNA-mediated *Sirt1* knockdown did not change *tPA, Ide*, and *Lrp1*, or the genes involved in β-amyloid production. However, *Sirt1* knockdown blocked the *Lpc*-EV-induced upregulation of *Mmp-2, Nep*, and *Mmp-9*, although to a lesser extent (Fig. 5h; Supplementary Fig. 4d). These results suggest that *Lpc*-EV restores the Aβ42-induced downregulation of *Mmp-2*, *Mmp-9*, and *Nep* through the upregulation of *Mecp2* and *Sirt1*.

### *Lpc*-EV treatment tended to suppress gliosis in the brains of Tg-APP/PS1 mice

**Fig. 6:**
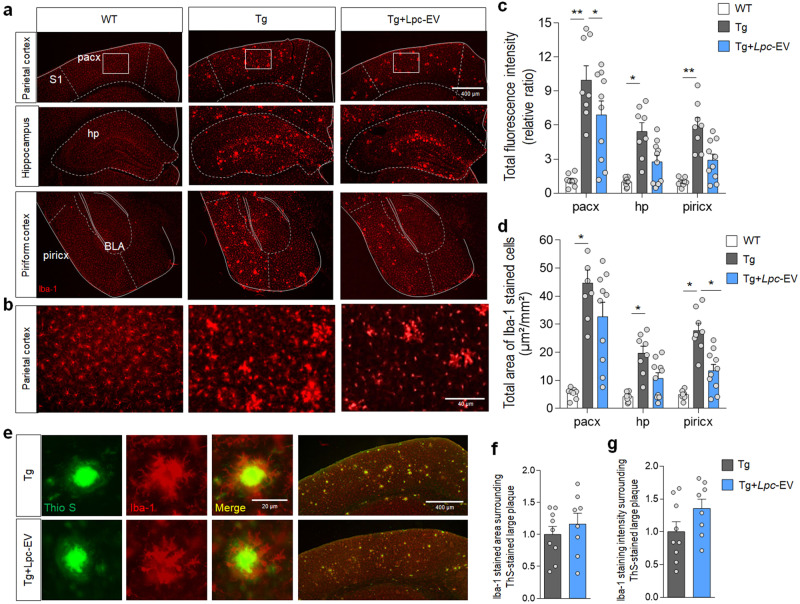
*Lpc*-EV treatment reduced microgliosis in the brains of Tg-APP/PS1 mice. **a**–**d** Photomicrographs showing the anti-Iba-1-stained parietal cortex (Pacx), dorsal hippocampus (dHP), and piriform cortex (Piricx) of wild-type mice (WT), Tg-APP/PS1 mice (Tg), and Tg-APP/PS1 mice treated with *Lpc*-EV (Tg+*Lpc*-EV) (**a**). Higher magnification of the boxed areas in the parietal cortex of the indicated groups (**b**). Relative ratio of total anti-Iba-1-stained fluorescent intensity (**c**) and total area of anti-Iba-1-stained cells (**d**) in the parietal cortex, dorsal hippocampus, and piriform cortex of the indicated groups. S1, somatosensory cortex 1; BLA, basolateral nucleus of amygdala. *n* = 8 animals for WT and Tg and 11 for Tg+*Lpc*-EV. Total intensity; F(2,23) = 16.17, *p* < 0.0001; F(2,23) = 13.85, *p* = 0.0001; F(2,23) = 19.43, *p* < 00.0001. Total anti-Iba-1-stained area; pacx, F(2,23) = 20.66, *p* < 00.0001; hp, F(2,23) = 14.78, *p* < 00.0001; piricx, F(2,23) = 28.27, *p* < 00.0001. **e**–**g** Photomicrographs (**e**) showing Iba-1-stained areas of microglia (red) surrounding ThS-stained plaques (green) in the parietal cortex of Tg-APP/PS1 mice (Tg) and Tg-APP/PS1 mice treated with Lpc-EV (Tg+*Lpc*-EV). Relative ratio of Iba-1-stained areas (**f**) and the intensity of Iba-1-stained microglia (**g**) over plaque areas. *n* = 8–9 animals. Iba-1-stained area ratio; t(15) = 0.7881, *p* = 0.4429; Iba-1-stained intensity ratio; t(15) = 1.677, *p* = 0.1143. Data are presented as mean ± SEM. *p < 0.05; **p < 0.01 (Student’s *t*-test; and one-way ANOVA followed by the Newman‒Keuls post hoc test).

Immunohistochemical analysis showed that compared to wild-type mice, Tg-APP/PS1 mice at 8 months of age had increased Iba-1 staining in microglia throughout the brain. *Lpc*-EV treatment in Tg-APP/PS1 mice tended to reduce Iba-1 staining in microglia compared to that in Tg-APP/PS1 controls, but the reduction in the parietal cortex and hippocampus was not statistically significant (Fig. 6a–d). Quantitative analysis indicated that the levels of Iba-1-stained microglia surrounding large plaques were not decreased following *Lpc*-EV treatment (Fig. 6e–g). In addition, GFAP staining indicated that Tg-APP/PS1 mice had increased astrogliosis throughout the brain, whereas Tg-APP/PS1 mice treated with Lpac-EV had significantly reduced levels of astrogliosis in the brain (Supplementary Fig. 5).

### *Lpc*-EV treatment increased neurogenesis in the hippocampus of Tg-APP/PS1 mice

**Fig. 7:**
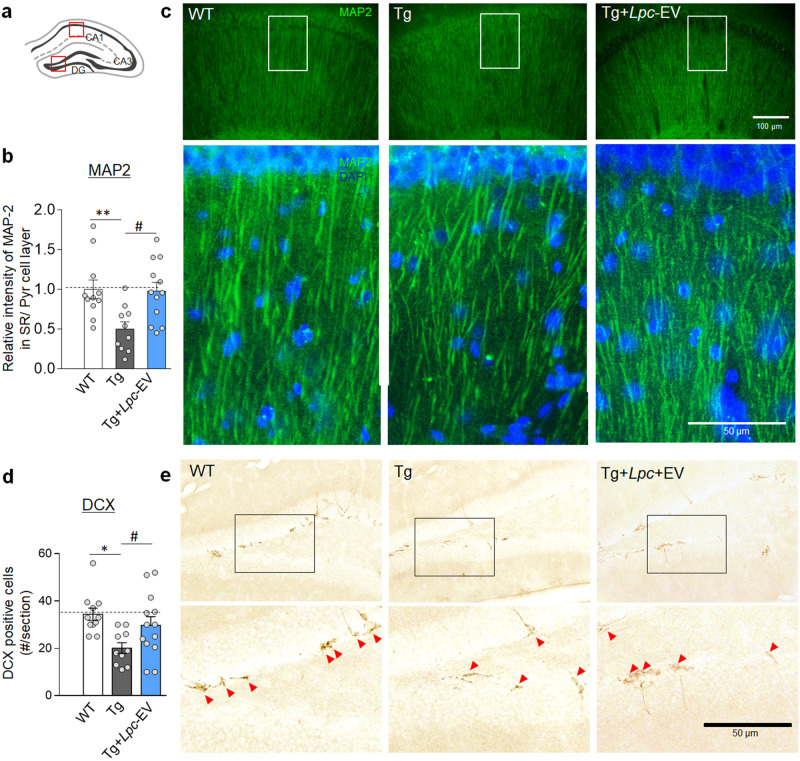
*Lpc*-EV treatment increased neurogenesis and MAP-2-stained density of hippocampal dendritic processes in Tg-APP/PS1 mice. **a**–**c** Diagram of the regions examined for immunohistochemical analyses (**a**). Anti-MAP2 staining levels in the stratum radiatum of the indicated groups (**b**). Photomicrographs showing anti-MAP2-stained dendritic processes of pyramidal neurons in the stratum radiatum (**c**) in the CA1 region of WT-CON, Tg-CON and Tg-*Lpc*-EV mice. Higher magnification (low panels) of the boxed areas (**c**) of the indicated groups. The red box in the CA1 of (**a**) is the region for the images in (**c**) (upper panels). *n* = 9–12 animals. F(2,30) = 6.385, *p* = 0.0049. **d**, **e** The numbers of anti-doublecortin (DCX)-positive cells in the dentate gyrus (DG) of WT-CON, Tg-CON, and Tg-*Lpc*-EV mice (**d**). Photomicrographs showing anti-doublecortin **(**DCX)-stained cells in the dentate gyrus of the indicated groups (**e**). The red box in the DG of (**a**) is the region for the images in (**e**) (upper panels). Higher magnification (lower panels) of the boxed areas (**e**) of the indicated groups. *n* = 10–13 animals. F(2,31) = 5.136, *p* = 0.0118. Data are presented as the mean ± SEM. *, **, #, difference between the indicated groups. *, #, *p* < 0.05; **p < 0.01 (one‐way ANOVA followed by Newman‒Keuls post hoc test).

The relative MAP2 staining level in the dendritic processes of CA1 pyramidal neurons compared to that in the cell body region of CA1 pyramidal neurons in the stratum radiatum in Tg-APP/PS1 mice was reduced compared to that in the stratum radiatum of wild-type mice. In contrast, compared to Tg-APP/PS1 mice, Tg-APP/PS1 mice treated with *Lpc*-EV had increased MAP2 staining levels in the dendritic processes of CA1 pyramidal neurons (Fig. 7a–c).

Tg-APP/PS1 mice at 8 months of age had fewer Ki-67 (a marker of proliferating cells)-positive cells and doublecortin (DCX; a marker of neuronal differentiation)-positive cells in the dentate gyrus compared than wild-type mice. In contrast, Tg-APP/PS1 mice treated with *Lpc*-EV had more Ki-67-positive cells and DCX-positive cells than Tg-APP/PS1 mice (Fig. 7d, e; Supplementary Fig. 6).

### *Lpc*-EV treatment rescued cognitive deficits in Tg-APP/PS1 mice

**Fig. 8:**
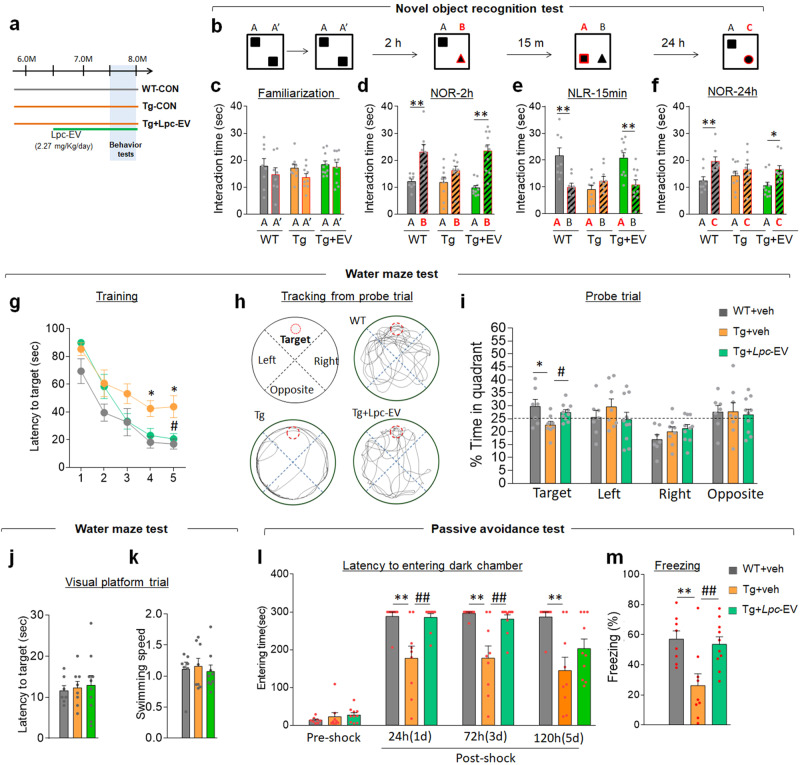
*Lpc*-EV treatment improved cognitive deficits in Tg-APP/PS1 mice. **a** Experimental design. Tg-APP/PS1 mice were orally administered *Lpc*-EV at a dose of 2.27 mg/kg/day from 6.5 months of age (green line) until the end of the behavioral tests. Behavioral tests were performed in the order of the novel object recognition test (NOR), water maze test (WM), and passive avoidance test (PA). **b**–**f** The novel object recognition test: The experimental steps in the NOR test (**b**). Time spent exploring between two identical objects during familiarization (**c**, familiarization), between a novel and a familiar object 2 h after familiarization (**d**, NOR-2 h), between a displaced object and a familiar object 15 min later (**e**, NLR-15 min), and between a novel and a familiar object 24 h after familiarization (**f**, NOR-24 h) for the indicated groups. *n* = 8, 9, and 10 for WT, Tg, and Tg+*Lpc*-EV, respectively. Familiarization; WT, t(14) = 0.8347, *p* = 0.4179; Tg, t(16) = 1.720, *p* = 0.1047; Tg+*Lpc*-EV, t(18) = 0.4314, *p* = 0.6713. NOR-2 h; WT, t(14) = 3.733, *p* = 0.0022; Tg, t(16) = 1.686, *p* = 0.1111; Tg+*Lpc*-EV, t(18) = 5.516, *p* < 0.0001. NLR-15 min; WT, t(14) = 3.557, *p* = 0.0032; Tg, t(16) = 1.283, *p* = 0.2178; Tg+*Lpc*-EV, t(18) = 3.561, *p* = 0.0022. NOR-24 h; WT, t(14) = 3.210, *p* = 0.0063; Tg, t(16) = 0.8527, *p* = 0.4064; Tg+*Lpc*-EV, t(18) = 2.753, *p* = 0.0131. **g**–**k** The water maze test: The latency to find the hidden platform in the hidden platform trial (**g**) for the indicated groups. Representative tracking (**h**) and time spent (**i**) in each quadrant in the probe trial of the indicated groups. The dashed line indicates a 25% chance of exploring a quadrant. C, center; P, periphery; T, target; L, left; R, right; O, opposite. The latency to find the platform during the visual platform trial (**j**) and swim speed (**k**) in the visual platform trial of the indicated groups. *n* = 7 (WT), 8 (Tg), and 10 (Tg+*Lpc*-EV). Training (**g**); genotype, F(2,22) = 6.026, *p* = 0.0079; *Lpc*-EV treatment, F(4,88) = 51.43, *p* < 0.0001; interaction, F(8,88) = 1.531, *p* = 0.1572. Probe trial (**i**); target, F(2,22) = 4.479, *p* = 0.0233. Latency (**j**), F(2,21) = 0.2587, *p* = 0.7742; Speed (**i**), F(2,21) = 0.1366, *p* = 0.8731. **l**, **m** The passive avoidance test: the latency to enter the dark chamber at the preshock, and 24 h, 72 h, and 120 h after shock (**l**), and the freezing time 24 h after shock (**m**) for the indicated groups. *n* = 7 (WT), 8 (Tg), and 10 (Tg+*Lpc*-EV). Preshock, F(2,24) = 0.7906, *p* = 0.4650; 24 h, F(2,24) = 9.480, *p* = 0.0009; 72 h, F(2,24) = 10.23, *p* = 0.0006; 120 h, F(2,24) = 6.479, *p* = 0.0056; freezing time, F(2,24) = 7.227, *p* = 0.0035. Data are presented as the mean ± SEM. *p < 0.05; **p < 0.01, difference between indicated groups; #*p* < 0.05; ##*p* < 0.01, difference between Tg and Tg-*Lpc*-EV (Student’s *t*-test; one-way ANOVA followed by the Newman‒Keuls post hoc test; and two-way repeated-measures ANOVA followed by the Bonferroni post hoc test).

Next, we investigated whether *Lpc*-EV treatment improves cognitive deficits in Tg-APP/PS1 mice (Fig. 8a). In the novel object recognition (NOR) test (Fig. 8b), wild-type mice, Tg-APP/PS1 mice, and Tg-APP/PS1 mice treated with *Lpc*-EV explored two identical objects for a similar amount of time during the familiarization phase (Fig. 8c). In the NOR test two hours later (NOR-2 h), Tg-APP/PS1 mice did not prefer the novel object over the familiar object, whereas Tg-APP/PS1 mice treated with *Lpc*-EV preferentially explored the novel object over the familiar object (Fig. 8d). In the novel location recognition test performed 15 min later (NLR-15 min), Tg-APP/PS1 mice did not prefer the displaced object, whereas Tg-APP/PS1 mice treated with *Lpc*-EV explored the displaced object over the novel object presented 15 min before (Fig. 8e). In the NOR test examined 24 h after familiarization (NOR-24 h), Tg-APP/PS1 mice did not preferably explore the novel object over the familiar object. In contrast, Tg-APP/PS1 mice treated with *Lpc*-EV preferentially explored the novel object over the familiar object (Fig. 8f). These results suggest that Tg-APP/PS1 mice treated with *Lpc*-EV show improved object recognition and retention memory.

Compared to wild-type mice, Tg-APP/PS1 mice exhibited an increase in latency to find the hidden platform compared to wild-type mice during the training phase of the water maze test. Tg-APP/PS1 mice treated with *Lpc*-EV showed a shortened latency to find the hidden platform compared to that of Tg-APP/PS1 control mice from Days 4 and 5 (Fig. 8g). In the following probe trial in which the escape platform was removed, Tg-APP/PS1 mice showed reduced exploration time in the target quadrant compared to that of wild-type mice, whereas Tg-APP/PS1 mice treated with *Lpc*-EV showed increased exploration time in the target quadrant compared to that of Tg-APP/PS1 mice (Fig. 8h, i). In the visual platform trial, wild-type mice, Tg-APP/PS1 mice, and Tg-APP/PS1 mice treated with *Lpc*-EV used similar amounts of times to reach the visual platform and similar swimming speeds during the test trial (Fig. 8j, k).

Compared to wild-type mice, Tg-APP/PS1 mice exhibited a reduced latency to enter the shock-associated dark chamber examined at 24 h, 72 h and 120 h after shock and reduced freezing time in the light chamber at 24 h after shock during the passive avoidance test. In contrast, Tg-APP/PS1 mice treated with *Lpc*-EV showed an increased latency to enter the shock-associated chamber until 72 h after shock and increased freezing time at 24 h after shock compared to that of Tg-APP/PS1 mice, suggesting that *Lpc*-EV treatment improved shock-associated retention memory (Fig. 8l, m).

## Discussion

### *Lpc*-EV counteracted the Aβ-induced reduced expression levels of neurotrophic factors and TrkB through the upregulation of epigenetic factors

In the present study, we demonstrated that *Lpc*-EV treatment in HT22 cells upregulated the expression of *Bdnf, Nt3, Nt4/5*, and *TrkB*, along with upregulating or downregulating a number of nuclear or epigenetic factors, including *Mecp2, Sirt1*, and *Hdac2* (Figs. 1, 2). Using systematic siRNA-mediated knockdown experiments, we provided evidence that *Lpc*-EV upregulated the expression of *Bdnf, Nt3, Nt4/5*, and *TrkB* through the transcriptional activation of those epigenetic factors (Fig. 3; Supplementary Figs. 2, 3). The results of the present study suggest the following interrelated issues regarding the roles of epigenetic factors in mediating *Lpc*-EV effects.

First, *Lpc*-EV upregulated the expression of *Bdnf, Nt3, Nt4/5*, and *TrkB* through activation of multiple epigenetic factors. Of the epigenetic factors, *Mecp2* and *Sirt1* were critical players in *Lpc*-EV-induced upregulation of *Bdnf, Nt3, Nt4/5*, and *TrkB* (Fig. 3a, b), whereas *Sirt5, Kdm4a, Hdac2*, and *G9a* were partially or selectively involved in regulating the expression of *Bdnf, Nt3, Nt4/5*, and *TrkB*. The identified epigenetic factors were organized to form a hierarchical regulatory network, while partially converging onto *Mecp2* and *Sirt1* (Supplementary Fig. 7). Although the bioactive components of *Lpc*-EV remain unknown, it is possible that *Lpc*-EV cargo contains multiple types of bioactive components that activate several nodes in the regulatory network of the epigenetic factors (Supplementary Fig. 7). Considering that bacterial EVs contain various proteins, chemical metabolites, fatty acids, and nucleic acids^43–45^, candidate bioactive *Lpc*-EV cargo contents could be such bacterial components. Second, *Lpc*-EV counteracted Aβ42-induced pathological changes. *Lpc*-EV treatment in HT22 cells changed the expression of *Bdnf*, *Nt3*, *Nt4/5*, *TrkB*, *Mecp2, Sirt1, Sirt5, Kdm4a, Hdac2*, and *G9a* in opposite directions to those induced by Aβ42 (Fig. 1a–f; Figs. 2, 3). It is worthwhile to understand the underlying mechanisms of how the two unrelated materials, Aβ42 and *Lpc*-EV, modulate the expression of the same epigenetic factors in opposite directions, but they remain to be elucidated. Third, our statistical analysis of *Lpc*-EV effects on HT22 cells indicates that *Lpc*-EV increased the transcript levels of *Bdnf, Nt3, Nt4/5, Ngf, TrkB*, *Mecp2, Creb1, Sirt1*, and *Sirt5*, and decreased the transcript levels of *Setdb1, G9a*, and *Kdm4a* (Fig. 1). These results raise the possibility that *Lpc*-EV can be used in the treatment of other brain disorders that are proceeded by reduced expression of neurotrophic factors, *TrkB, Mecp2*, and *Sirt1. Lactobacillus plantarum*-derived EVs (*Lpl*-EV) has the ability to counteract the stress-induced downregulation of neurotrophic factors, *MeCP2*, and *Sirt1* in the brain and improves depressive-like behavioral deficits^10,11^. It remains to be determined whether *Lpc*-EV can exert protective effects against stress-induced depression-like behaviors, similar to those of *Lpl*-EV.

MeCP2- and Sirt1-dependent mechanisms in the regulation of neurotrophic factor expression have been reported in various cellular contexts. MeCP2 knockout mice have downregulated expression of *Bdnf* in the hippocampus^20,21^. Tg-APP/PS1 mice have reduced expression levels of neurotrophic factors and MeCP in the hippocampus^22^. Consistently, MeCP2 binding to the promoter of the *Bdnf, Nt3*, and *Nt4/5* genes is positively correlated with the expression levels of *Bdnf, Nt3*, and *Nt4/5* in the hippocampus of Tg-APP/PS1 mice, and siRNA-mediated *Mecp2* knockdown in the hippocampus downregulated the expression of *Bdnf, Nt3*, and *Nt4/*5 in the injected site^11^. Sirt1 regulates MeCP2 by deacetylation, which leads to an increase in *Bdnf* expression levels^46^. Sirt1 also upregulates *Bdnf* expression in the hippocampus in a CREB-dependent manner^47^. In the present study, *Mecp2* or *Sirt1* knockdown blocked the *Lpc*-EV-induced upregulation of *Bdnf, Nt3, Nt4/5*, and *TrkB* in HT22 cells, whereas *Mecp2* or *Sirt1* knockdown produced subtle or no effect on *Creb*, respectively (Fig. 3f, h). Although the present study demonstrated the importance of MeCP2- and Sirt-1-dependent mechanisms in mediating *Lpc*-EV effects, it is possible that the epigenetic mechanisms regulating *Lpc*-EV effects on the brains of Tg-APP/PS1 mice are more complex than those in HT22 cells. In addition, considering that the regulatory network composed of multiple epigenetic factors (Supplementary Fig. 7) might have a role in integrating other signaling pathways in neuronal and nonneuronal cells, we cannot rule out the possibility that MeCP2- or Sirt1-independent pathways have a role in regulating *Lpc*-EV effects.

### Possible mechanisms underlying the *Lpc*-EV-induced alleviation of AD-like pathology in the brains of Tg-APP/PS1 mice

*Lpc*-EV treatment in Tg-APP/PS1 mice alleviated key AD-like pathology, including Aβ-plaque deposition (Fig. 5), neuroinflammatory responses (Fig. 6; Supplementary Fig. 5), and neurogenesis (Fig. 7; Supplementary Fig. 6), and improved cognitive deficits (Fig. 8). Although the detailed mechanism by which *Lpc*-EV produces such diverse effects in Tg-APP/PS1 mice needs to be further characterized, our results raise the following possibilities.

First, the *Lpc*-EV-induced upregulation of neurotrophic factors and TrkB might contribute to increased neurogenesis, neuroprotection of dendritic morphology, and enhanced cognitive function in Tg-APP/PS1 mice. BDNF regulates synaptic and behavioral plasticity^48–51^. However, the available evidence shows conflicting results. Viral vector-mediated BDNF expression in the entorhinal cortices improved hippocampal-dependent contextual fear conditioning, but it did not reduce Aβ accumulation in transgenic mice (J20 strain) that carry APP Indiana (V717F) and Swedish (K670M) mutations^52^. In contrast, the BDNF deficiency increased Aβ production and BDNF overexpression counteracted the downstream consequences of Aβ accumulation^53,54^. Therefore, it is worthwhile to study whether the *Lpc*-EV-induced upregulation of neurotrophic factors and TrkB modifies Aβ accumulation.

Second, the *Lpc*-EV-induced upregulation of *Mmp-2*, *Mmp-9*, and *Nep* might play a role in mitigating Aβ accumulation in the brains of Tg-APP/PS1 mice (Fig. 5a–e). *Lpc*-EV counteracted Aβ42-induced downregulation of *Mmp-2*, *Mmp-9*, and *Nep* in HT22 cells (Fig. 5f). In contrast, siRNA-mediated knockdown of *Mecp2* or *Sirt1* blocked *Lpc*-EV-dependent upregulation of *Mmp-2*, *Mmp-9*, and *Nep* (Fig. 5g, h). These results are consistent with a recent report that siRNA-mediated *Mecp2* knockdown downregulates *Mmp-2* and *Mmp-9* expression^55^. Our results support the notion that *Lpc*-EV treatment produces MeCP2- and Sirt1-dependent upregulation of *Mmp-2*, *Mmp-9*, and *Nep* (Fig. 5f–h), which in turn reduces Aβ42 accumulation and improves neuroinflammatory responses and other AD-like pathology.

Third, the decreased neuroinflammatory responses induced by *Lpc*-EV might reduce Aβ pathology in the brains of Tg-APP/PS1 mice. Chronic neuroinflammation increases Aβ plaque deposition^56,57^, decreases hippocampal neurogenesis^58^, and induces cognitive decline^59,60^. Recently, we reported that *Lpc*-EV treatment in HT-29 human colorectal cancer cells downregulates the expression of LPS-induced pro-inflammatory cytokines IL-1α, IL-1β, IL-2, and TNFα; increases the expression of the anti-inflammatory cytokines IL-10 and TGFβ; and attenuates intestinal inflammatory responses in dextran sulfate sodium (DSS)-induced colitis in C57BL/6 mice^12^. Therefore, it is possible that the *Lpc*-EV-induced suppression of neuroinflammatory responses contributes to improving pathological deficits in the brains of Tg-APP/PS1 mice, although direct evidence should be provided.

In conclusion, our results suggest that *Lpc*-EV has the ability to induce MeCP2- and Sirt1-dependent upregulation of *Bdnf*, *Nt3*, *Nt4/5*, *TrkB*, *Mmp-2* and *Mmp-9*, and epigenetic modification is a critical mechanism by which *Lpc*-EV alleviates AD-like pathology in Tg-APP/PS1 mice.

### Supplementary information

Supplemental Figures

## Data Availability

Data and materials will be made available on reasonable request.
