# Supplementary material for: Lactobacillus-derived extracellular vesicles counteract Aβ42-induced abnormal transcriptional changes through the upregulation of MeCP2 and Sirt1 and improve Aβ pathology in Tg-APP/PS1 mice
Source: Exp Mol Med. 2023 Sep 13;55(9):2067–82. doi: 10.1038/s12276-023-01084-z (PMC10545704; doi:10.1038/s12276-023-01084-z)
Supplement: Supplementary file 1 — Supplemental Figures [file 12276_2023_1084_MOESM1_ESM.pdf]

SUPPLEMENTAL INFORMATION

Lactobacillus-derived extracellular vesicles counteract Aβ42-induced abnormal transcriptional changes through the upregulation of MeCP2 and Sirt1 and improve Aβ pathology in Tg-APP/PS1 mice

Kwon, H. et al.,

Corresponding authors: Dr. YK Kim (e-mail: ykkim@mdhc.kr), and
Dr. PL Han (e-mail: plhan@ewha.ac.kr)

Illustrations:

Supplementary Fig. 1-7 and figure legends

Supplementary Fig. 1

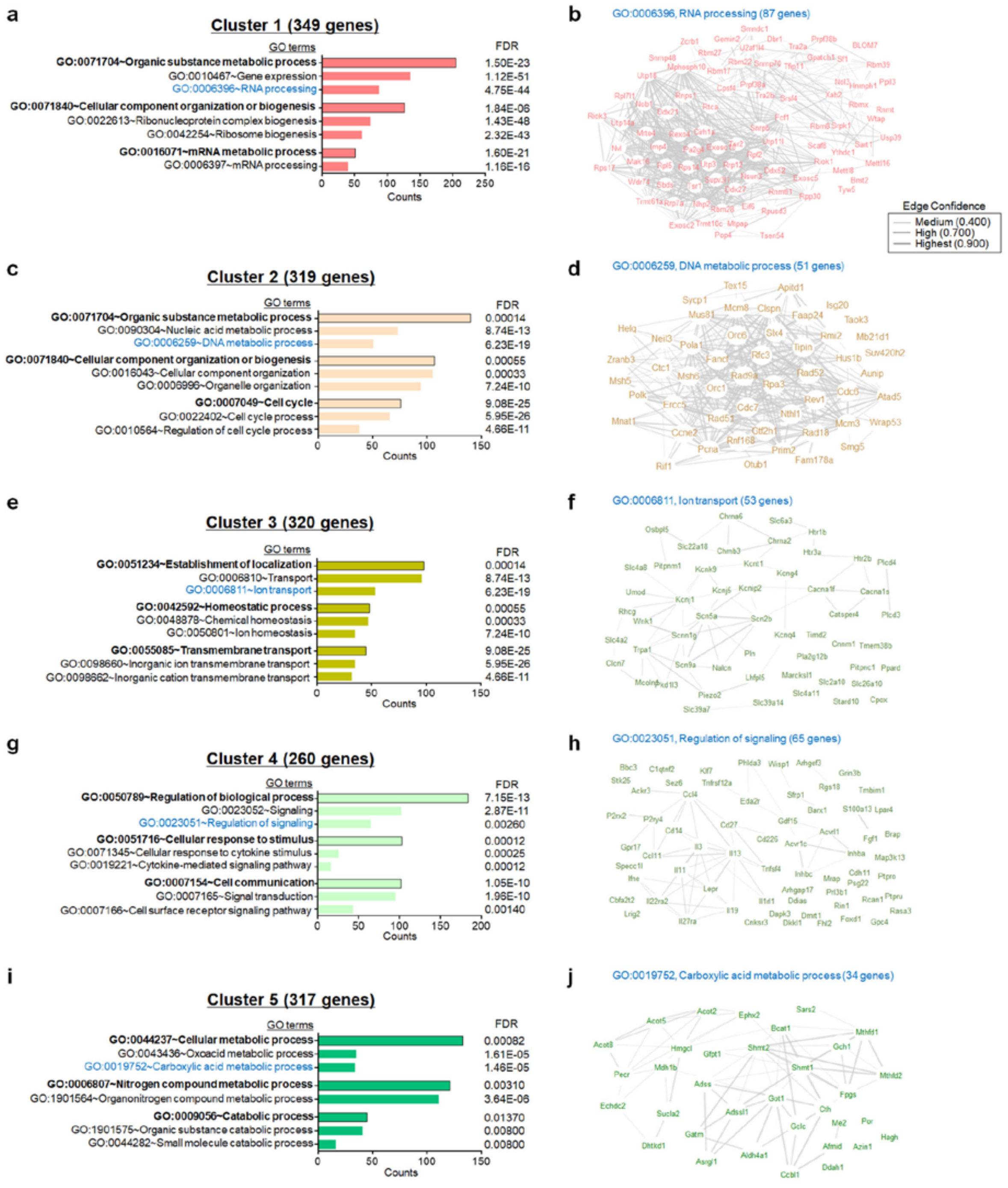

Supplementary Fig. 1. Serial K-means clustering and Gene Ontology (GO) enrichment analyses identified seven functional clusters of genes with a wide range of biological functions.

a-j, A summary of key features of identified functional clusters. Top three functional groups with specific biological processes represented by GO term and PPI of a selected subgroup in each cluster were presented; Cluster # 1 contained genes involved in “Organic substance metabolic process”, which included factors involved

in RNA processing”, and “Cellular component organization or biogenesis” (**a, b**); Cluster #2 contained genes involved in “Organic substance metabolic process”, which included factors involved in DNA metabolic process, and “Cell cycle” (**c, d**); Cluster #3 contained genes involved in “Establishment of localization”, which included factors involved in ion transport, and “Homeostatic process” (**e, f**); Cluster #4 contained genes involved in “Regulation of biological process”, which included factors involved in regulation of signaling, and “Cell communication” (**g, h**); and Cluster #5 contained genes involved in “Cellular metabolic process”, which included factors involved in carboxylic acid metabolic process, and “Nitrogen compound metabolic process” (**i, j**). Clusters #6 and #7 were presented in Fig. **2e-h**.

Supplementary Fig. 2

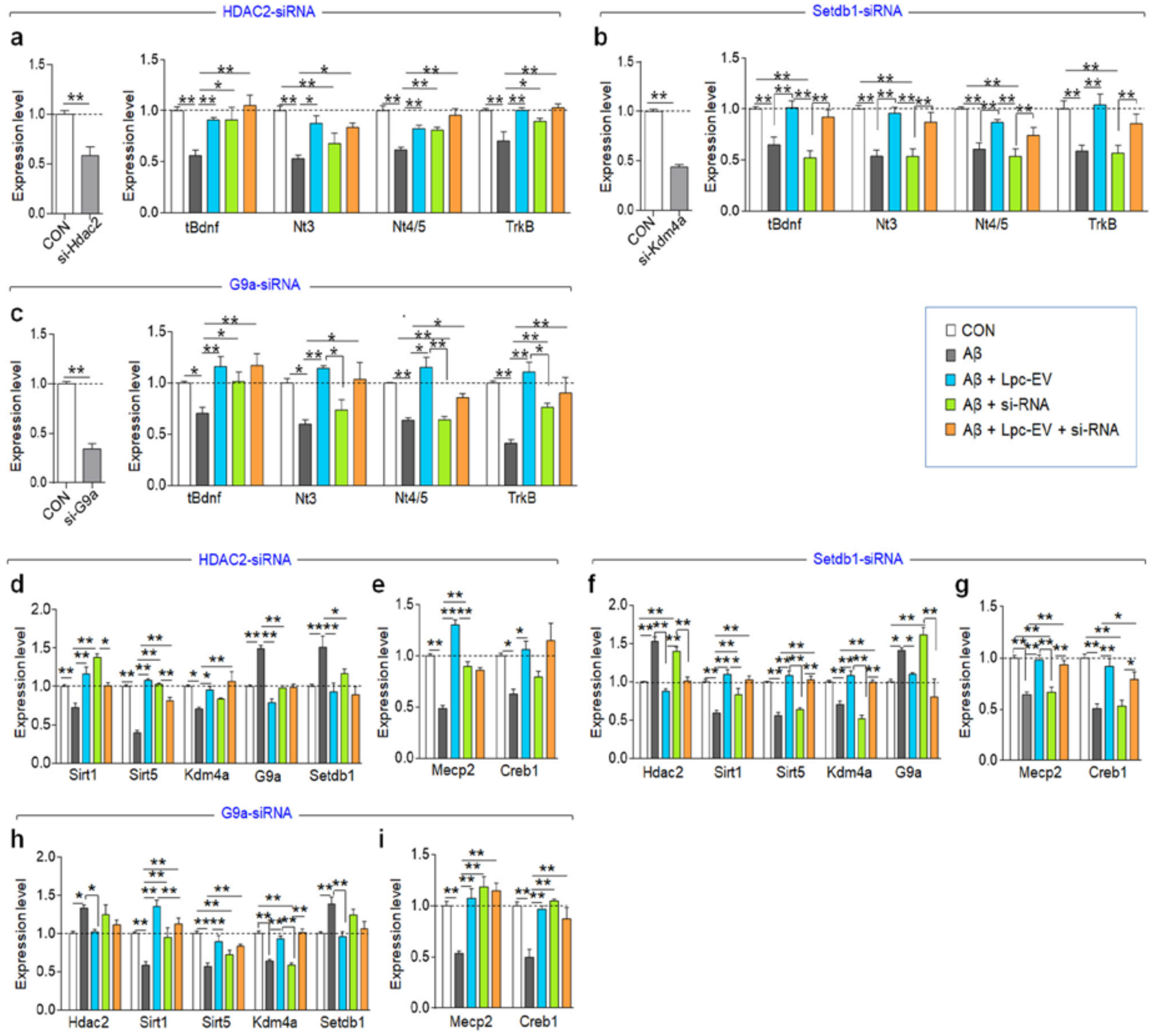

Supplementary Fig. 2. *Lpc*-EV counteracted the Aβ42-induced upregulation of neurotrophic factors and *TrkB* via the downregulation of multiple epigenetic factors in HT22 cells.

a-c Expression levels of *Bdnf*, *Nt3*, *Nt4/5*, and *TrkB* in HT22 cells treated with Aβ42 (1 μM, final), Aβ42 plus *Lpc*-EV (10 μg/ml, final), Aβ42 plus the indicated siRNA, or Aβ42 plus *Lpc*-EV and the indicated siRNA: siRNA-Hdac2 (**a**), siRNA-Setdb1(**b**), and siRNA-G9a (**c**). siRNA-control, siCON.

d-i Expression of *Hdac2, Sirt1, Sirt5, Setdb1, Kdm4a, G9a*, *Mecp2,* and *Creb1* in HT22 cells treated with Aβ42 (1 μM), Aβ42 plus *Lpc*-EV (10 μg/ml), or Aβ42 plus the indicated siRNA: siRNA-Hdac2 (**d, e**), siRNA-Setdb1(**f, g**), and siRNA-G9a (**h, i**).

Data are presented as the mean ± SEM (n= 6 - 8). *, $p < 0.05$; **, $p < 0.01$ (Student's *t*-test; and one-way ANOVA followed by the Newman-Keuls *post hoc* test).

Supplementary Fig. 3

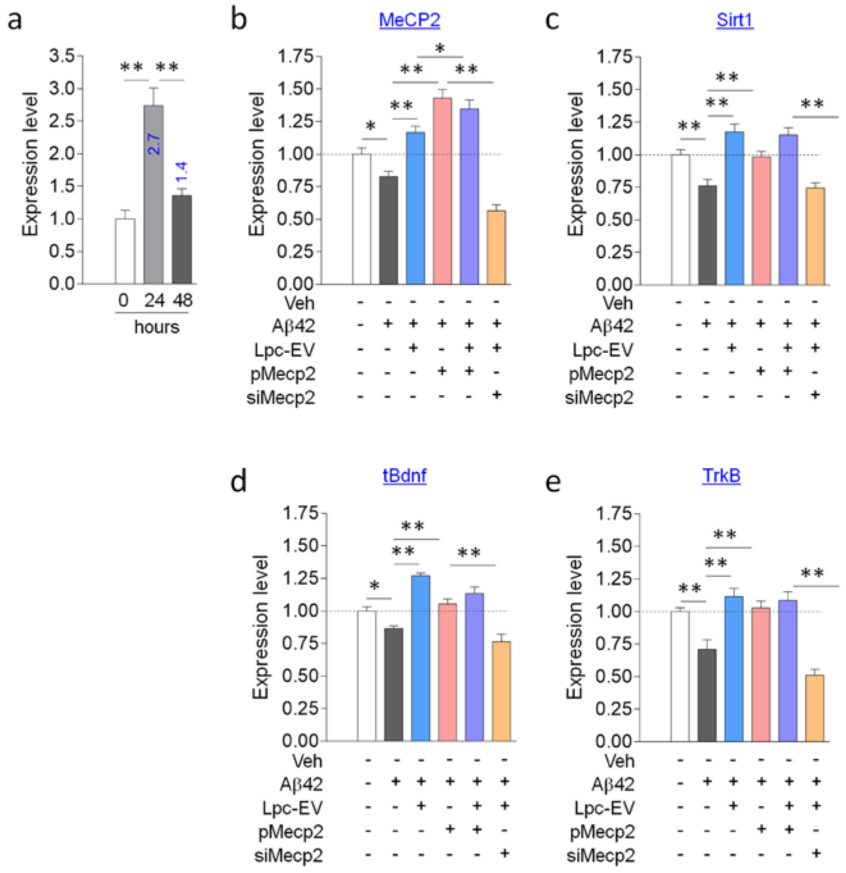

Supplementary Fig. 3. MeCP2 overexpression reversed Aβ42-induced downregulation of neurotrophic factors and TrkB in HT22 cells.

a-e MeCP2 transcript levels in HT22 cells after 24 h and 48 h of transient transfection of pCNS-D2-MeCP2 (pMecp2) (**a**). Twenty-four hours after transfection, cells were treated for 24 h with Aβ42 (1 μM, final), Aβ42 plus *Lpc*-EV (10 μg/ml, final), Aβ42 plus pMecp2, Aβ42 plus *Lpc*-EV plus pMecp2, or Aβ42 plus *Lpc*-EV plus siRNA-Mecp2. Treated cells were harvested and expression levels of *Mecp2* (**b**), *Sirt1* (**c**), total *Bdnf* (**d**), and *TrkB* (**e**) were analyzed using RT-PCR.

Data are presented as the mean ± SEM (n= 4, each group). *, $p < 0.05$; **, $p < 0.01$ (one-way ANOVA followed by the Newman-Keuls *post hoc* test).

Supplementary Fig. 4

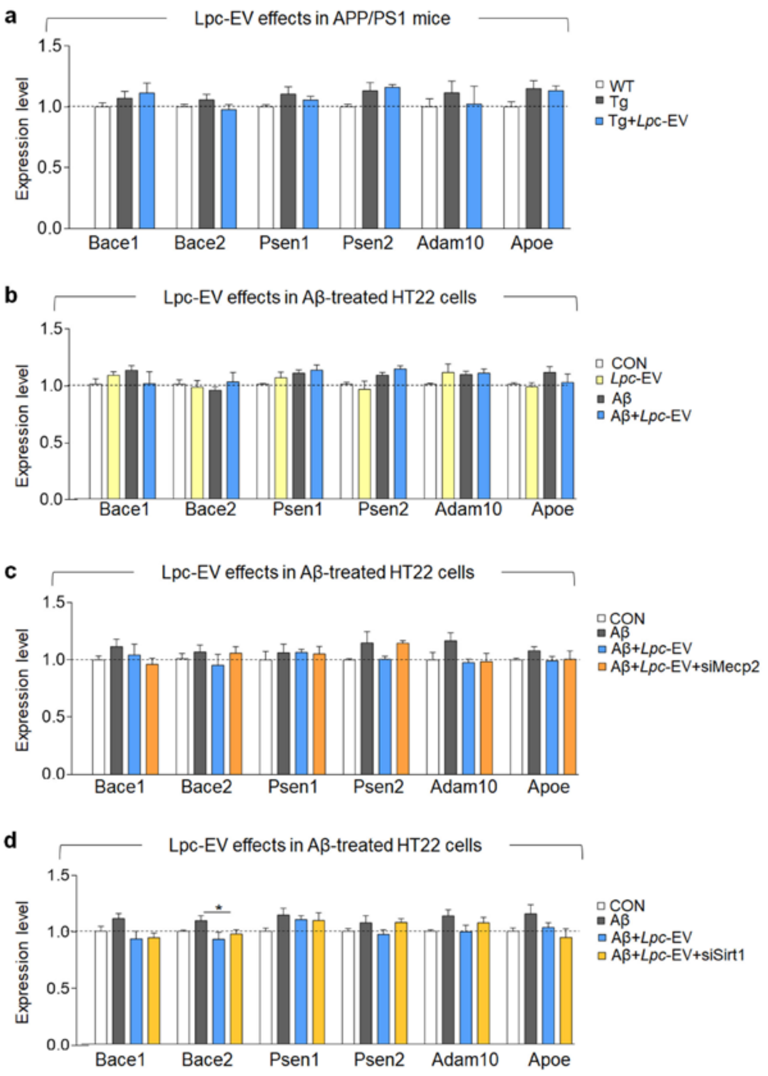

Supplementary Fig. 4. *Lpc*-EV treatment did not change the expression of Aβ42 producing genes in the hippocampus of Tg-APP/PS1 mice.

a Expression levels of *Bace1, Bace2, Psen1, Psen2, Adam10, Apoe* in the hippocampus of WT mice, Tg-APP/PS1 mice, and Tg-APP/PS1 mice treated with *Lpc*-EV. n = 8-9 animals per group, 6-8 PCR repeats.

b Expression levels of *Bace1, Bace2, Psen1, Psen2, Adam10, Apoe* in HT22 cells (CON), HT22 cells treated with *Lpc*-EV (10 μg/ml), HT22 cells treated with Aβ42 (1 μM), and HT22 cells treated with Aβ42 plus *Lpc*-EV. n = 6-8 PCR repeats.

c, d Expression levels of *Bace1, Bace2, Psen1, Psen2, Adam10, Apoe* in HT22 cells (CON), HT22 cells treated with Aβ42, and HT22 cells treated with Aβ42 plus the indicated siRNA; *siRNA*-MeCP2 (**c**) and *siRNA*-MeCP2 (**d**). n = 6-8 PCR repeats.

Data are presented as the mean ± SEM. $^{*}p < 0.05$ (one-way ANOVA followed by the Newman-Keuls *post hoc* test; and two-way ANOVA followed by the Bonferroni *post hoc* test).

Supplementary Fig. 5

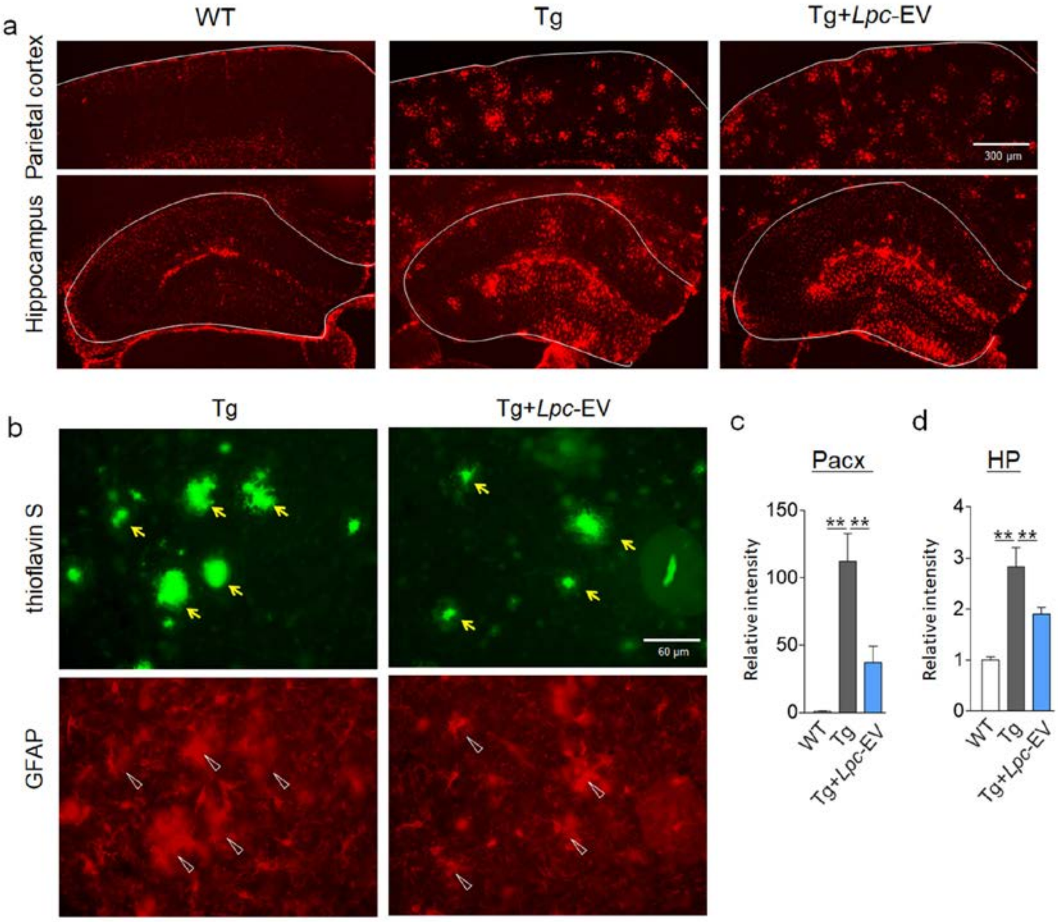

Supplementary Fig. 5. *Lpc*-EV treatment suppressed astrogliosis in the brain of Tg-APP/PS1 mice.

a-d Photomicrographs showing anti-GFAP-stained parietal cortex (Pacx) and dorsal hippocampus (dHP) (**a**) of wildtype mice (WT), Tg-APP/PS1 mice (Tg), and Tg-APP/PS1 mice treated with *Lpc*-EV (Tg+*Lpc*-EV). Higher magnification of plaque areas stained with thioflavin S (arrows) and associated with reactive astroglia (GFAP, arrow heads) (**b**) in the parietal cortex of Tg and Tg+*Lpc*-EV. Relative ratio of total anti-GFAP-stained fluorescent intensity in the parietal cortex (**c**) and dorsal hippocampus (**d**) of the indicated groups.

Data are presented as the mean ± SEM (n = 7 – 8 animals). $^*p < 0.05$ (one-way ANOVA followed by the Newman-Keuls *post hoc* test).

Supplementary Fig. 6

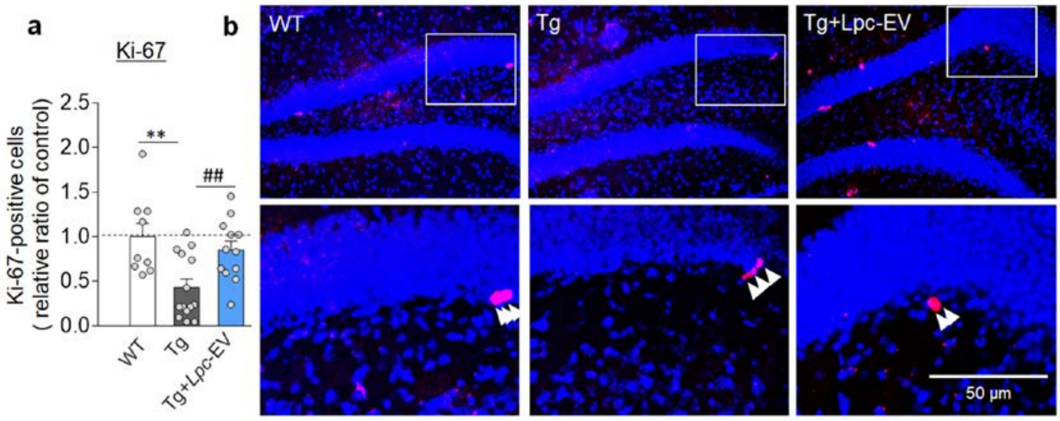

Supplementary Fig. 6. *Lpc*-EV treatment increased neurogenesis in Tg-APP/PS1 mice.

a,b Photomicrographs showing anti-Ki67-stained cells in the dentate gyrus (**b**) of WT-CON, Tg-CON and Tg-*Lpc*-EV mice. Higher magnification of the boxed areas of the indicated groups (lower panels). Quantification of anti-Ki67-positive cells in the dentate gyrus of the indicated groups (**a**).

Data are presented as the mean ± SEM (n = 9 - 12 animals). *,**, difference between the indicated groups. *, p<0.05; **, p<0.01 (one-way ANOVA followed by the Newman-Keuls *post hoc* test).

Supplementary Fig. 7

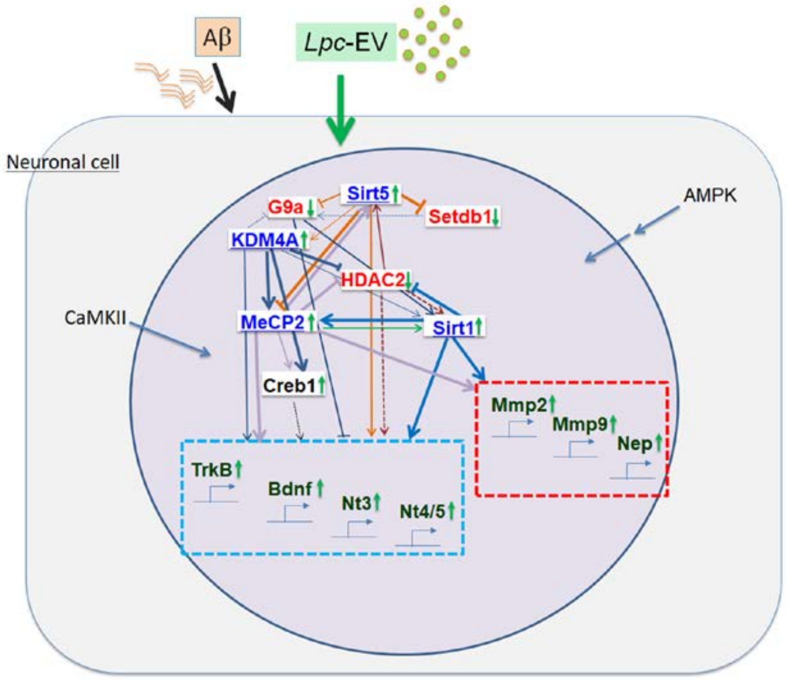

Supplementary Fig. 7: A regulatory network mediating *Lpc*-EV effects on the upregulation of neurotrophic factors and Aβ-degrading proteinases.

A hierarchical regulatory network consisting of multiple epigenetic factors activated by Aβ42- and *Lpc*-EV in opposite directions, and their target genes *Bdnf*, *Nt3*, *Nt4/5*, and *Trkb,* are grouped in the blue dotted box; *Mmp-2, Mmp-9,* and *Nep* are grouped in the red dotted box. Epigenetic factors are presented in red or blue letters when up- or down-regulated by Aβ42, respectively, and are also marked with red or blue short-arrows as their expression is up- or down-regulated by *Lpc*-EV. Fully effective and partially effective pathways involved in mediating *Lpc*-EV effects are marked with thick or thin lines, respectively, and weak but possible pathways are marked with dotted lines. MeCP2 and Sirt1 comprise the critical nodes of the regulatory network since their knockdown blocked *Lpc*-EV-induced upregulation of *Bdnf*, *Nt3*, *Nt4/5*, *TrkB, Mmp-2, Mmp-9*, and *Nep*.
